# Supplementary material for: Human inherited RORγT deficiency encompasses genetic heterogeneity, T cell deficiency, and clinical homogeneity
Source: medRxiv. 2026 Jul 20:2026.07.18.26358075. Preprint. [Version 1] doi: 10.64898/2026.07.18.26358075 (PMC13419669; doi:10.64898/2026.07.18.26358075)
Supplement: 1 [file NIHPP2026.07.18.26358075V1-supplement-1.pdf]

medRxiv preprint doi: https://doi.org/10.64898/2026.07.18.26358075; this version posted July 20, 2026. The copyright holder for this preprint (which was not certified by peer review) is the author/funder, who has granted medRxiv a license to display the preprint in perpetuity.
It is made available under a CC-BY 4.0 International license .

Supplemental Material

Fig. S1 supplements Fig. 1 and Fig. 2 and shows that V45F/V24F, K381I/K460I, Y186*/Y165*, T421Nfs*56/ T400Nfs*56, and R98*/R77* *RORC* variants are LOF when overexpressed, whereas common population *RORC* variants are functionally neutral. Fig. S2 supplements Fig. 3 and shows intact TCRα rearrangement heterozygous carriers of LOF *RORC* variants, as well as the patterns of IFN-γ and IL-17A production by lymphocyte subsets upon stimulation. Fig. S3 supplements Fig. 4 and shows deep immunophenotyping and longitudinal T-cell trends in patients with AR RORγ/RORγT deficiency. Fig. S4 supplements Fig. 5 and shows memory/exhaustion-associated skewing and distinct transcriptomic profiles in naïve T cells from patients with AR RORγ/RORγT deficiency. Fig. S5 supplements Fig. 7 and shows IFN-γ and TNF production by various lymphocyte subsets during mycobacterial infection.

medRxiv preprint doi: https://doi.org/10.64898/2026.07.18.26358075; this version posted July 20, 2026. The copyright holder for this preprint (which was not certified by peer review) is the author/funder, who has granted medRxiv a license to display the preprint in perpetuity.
It is made available under a CC-BY 4.0 International license .

Figure S1. Genome-wide analyses of RORγ/RORγT-deficient patients and functional characterization of the mutant and populational *RORC* variants in an overexpression system. (**A**) Schematic diagram depicting the workflow for the identification of the five new rare variants in *RORC.* (**B**) Sanger sequencing results to confirm the genotypes of P8 – P12 harboring new homozygous *RORC* variants. (**C**) Relative expression of *RORC* measured by quantitative RT-PCR (RT-qPCR) following the transfection of HEK293T cells with WT and mutant pCMV6-*RORC*-Myc-DDK plasmids. (**D**) Western-blot analysis of WT or mutant *RORC* isoform 1 (RORγ) variants to assess protein levels and subcellular distribution in HEK293T cells. C, cytoplasmic fraction; N, nuclear fraction. (**E**) Electromobility shift assay (EMSA) with a ^{32}P-labeled RORE-2 probe derived from the *IL17A* promoter, incubated with nuclear lysates of HEK239T cells transfected with the indicated vectors encoding RORγ, with or without PMA/ionomycin stimulation (P/I). (**F**) Transcriptional activity of patient RORγ variants in an *in vitro* luciferase reporter system driven by WT or mutant multimerized RORE. (**G** and **H**) Western-blot analysis of mutant *RORC* isoform 1 (RORγ) (G) and isoform 2 (RORγT) (H) variants from gnomAD to assess protein levels and subcellular distribution in HEK293T cells. C, cytoplasmic fraction; N, nuclear fraction. (**I**) Transcriptional activity of gnomAD variants shown in (G), as determined with an *in vitro* luciferase reporter system driven by WT or mutant multimerized RORE. All experimental data were verified in at least 2 independent experiments.

Figure S2. Normal TCRα rearrangement in heterozygous parents and similar levels of IL-17A production between patients and controls for certain lymphocyte subsets. (**A**) Bubble plot of high-throughput sequencing of the *TRAV/TRAJ* loci in genomic DNA from healthy controls and seven WT/M heterozygous carriers of loss-of-function *RORC* variants. Fold-change difference in *TRAV* and *TRAJ* usage in WT/M carriers relative to controls is indicated by bubble size, and statistical significance (*p*-value) by color, with *TRAV* segments on the *y*-axis and *TRAJ* segments on the *x*-axis. (**B and C**) Frequency of IL-17A-producing (B) and IFN-γ-producing cells (C) within the indicated subsets in

medRxiv preprint doi: https://doi.org/10.64898/2026.07.18.26358075; this version posted July 20, 2026. The copyright holder for this preprint (which was not certified by peer review) is the author/funder, who has granted medRxiv a license to display the preprint in perpetuity.
It is made available under a CC-BY 4.0 International license .

controls or patients, with or without P/I stimulation. All experimental data were verified in at least 2 independent experiments.

Figure S3. Deep immunophenotyping and trends over time in patients with AR RORγ/RORγT deficiency. (**A - G**) Frequencies of monocyte subsets (A), myeloid dendritic cell subsets (B), plasmacytoid dendritic cells (C), NK cell subsets (D), B-cell subsets (E), plasma cells (F), and ILC2 cells (G), among live PBMCs from controls, age-matched controls, and patients with AR IL-12Rβ1 or RORγ/RORγT deficiencies. (**H**) Longitudinal trend in the absolute T-cell counts of P11 relative to the reference range for age. (**I**) Frequencies of Vδ2^{+} γδ T cells among live PBMCs from controls, age-matched controls, and patients with AR IL-12Rβ1 or RORγ/RORγT deficiencies. (**J**) Gating strategy used for the identification of MAIT and iNKT cells. (**K - M**) Longitudinal trends in absolute CD4^{+} T-cell counts (K), absolute CD8^{+} T-cell counts (L), and frequencies of CD45RA^{+} CD4^{+} T cells (M) in P11 relative to the reference ranges for age. All experimental data were verified in at least 2 independent experiments.

Figure S4. Skewing toward memory and exhaustion-associated phenotypes and distinct transcriptomic profiles of naïve T cells from patients with AR RORγ/RORγT deficiency. (**A**) ImmuneSigDB C7 enrichment across pseudobulked immune subsets in RORγ/RORγT deficiency versus controls. Color indicates fold-change direction; size indicates -log10 false-discovery rate. (**B**) Focused module scores in naïve CD4^{+} and CD8^{+} T cells. Color indicates mean score difference between RORγ/RORγT deficiency and controls. All experimental data were verified in at least 2 independent experiments. (**C and D**) Heatmap of differentially regulated genes in naïve CD4^{+} T cells (C) and CD8^{+} (D) T cells across controls, IL-12Rβ1 deficiency, and RORγ/RORγT deficiency. (**E**) Canonical IFN/STAT1 gene-expression heatmap in naïve CD4^{+} and CD8^{+} T cells, used to assess whether STAT1-associated TF enrichment reflects type I or type II interferon biology.

medRxiv preprint doi: https://doi.org/10.64898/2026.07.18.26358075; this version posted July 20, 2026. The copyright holder for this preprint (which was not certified by peer review) is the author/funder, who has granted medRxiv a license to display the preprint in perpetuity.
It is made available under a CC-BY 4.0 International license .

Figure S5. Analysis of IFN-γ and TNF production during mycobacterial infection. (**A**) Production of IL-8, GM-CSF, and IL-1β by PBMCs with and without live BCG infection, in the presence or absence of exogenous IL-12 or IL-23. (**B**) UMAP visualization of leukocyte subsets in PBMCs from healthy controls, patients with IL-12Rβ1 deficiency, and patients with RORγ/RORγT deficiency. (**C and D**) Frequencies of TNF-producing cells among total lymphocytes (C), and among NK cells (D), following *in vitro* infection with live BCG, in the presence or absence of exogenous IL-12, IL-23, or IFN-γ. (**E and F**) Frequencies of IFN-γ-producing cells among $CD56^{dim}$ NK cells (E) and $CD56^{bright}$ NK cells (F), following *in vitro* infection with live BCG, in the presence or absence of exogenous IL-12, IL-23, or IFN-γ. (**G - J**) Frequencies of TNF-producing cells among $V\delta1^{+}$ γδ T cells (G), $V\delta2^{+}$ γδ T cells (H), $CD4^{+}$ T cells (I), and $CD8^{+}$ T cells (J), following *in vitro* infection with live BCG, in the presence or absence of exogenous IL-12, IL-23, or IFN-γ. All experimental data were verified in at least 2 independent experiments.

medRxiv preprint doi: https://doi.org/10.64898/2026.07.18.26358075; this version posted July 20, 2026. The copyright holder for this preprint (which was not certified by peer review) is the author/funder, who has granted medRxiv a license to display the preprint in perpetuity. It is made available under a CC-BY 4.0 International license .

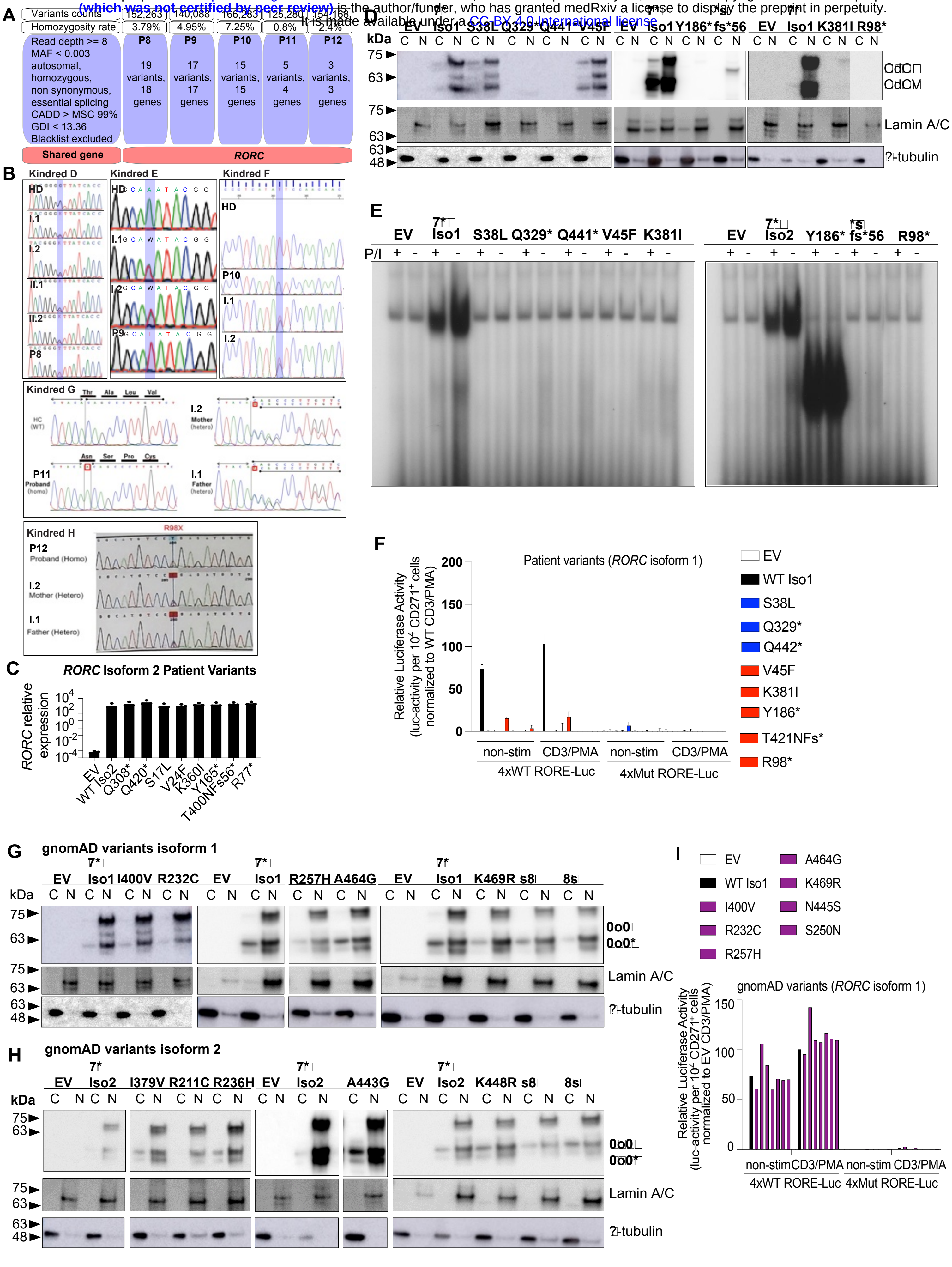

Fig S1

medRxiv preprint doi: https://doi.org/10.64898/2026.07.18.26358075; this version posted July 20, 2026. The copyright holder for this preprint (which was not certified by peer review) is the author/funder, who has granted medRxiv a license to display the preprint in perpetuity.
It is made available under a CC-BY 4.0 International license .

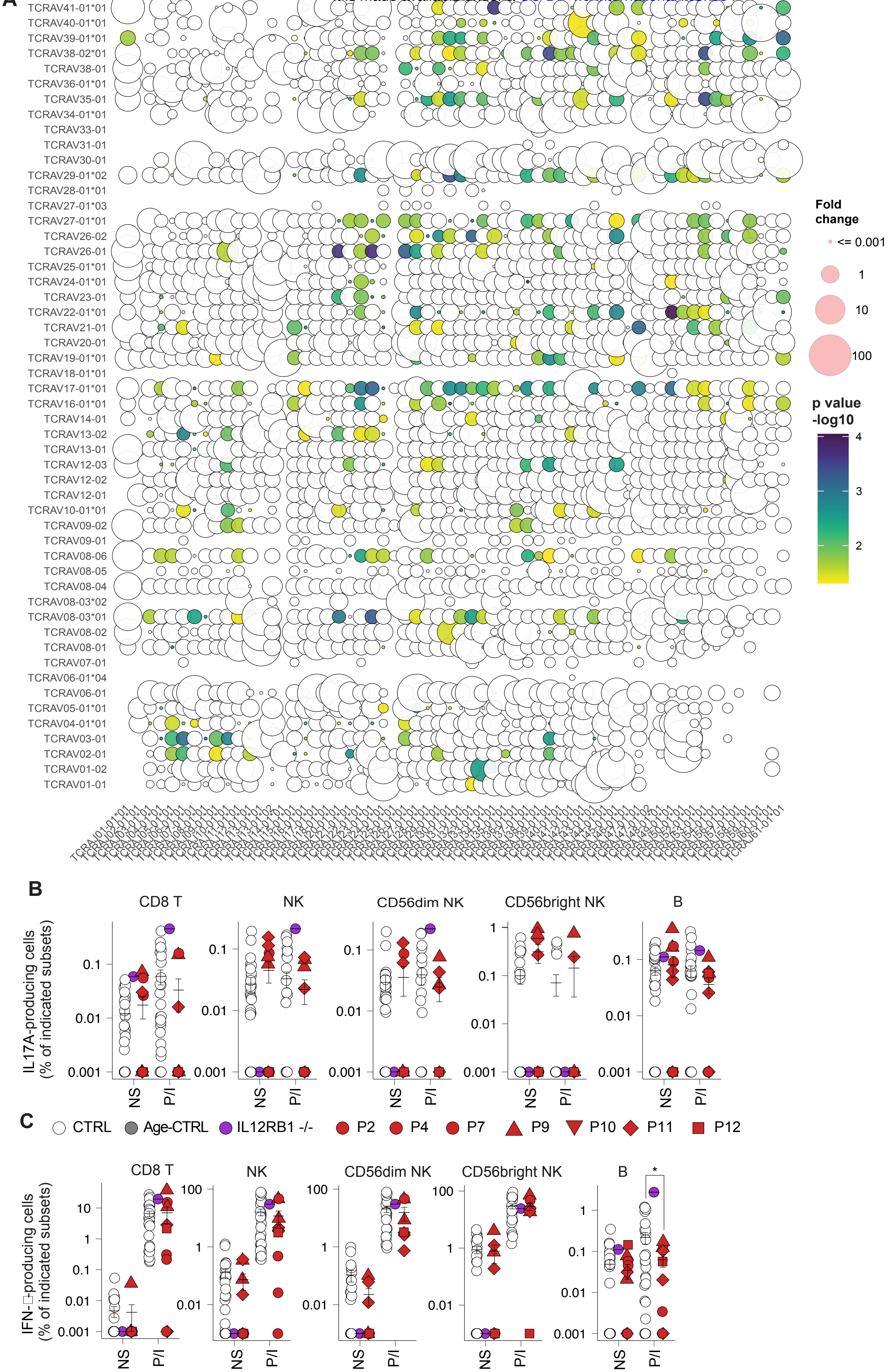

Fig S2

medRxiv preprint doi: https://doi.org/10.64898/2026.07.18.26358075; this version posted July 20, 2026. The copyright holder for this preprint (which was not certified by peer review) is the author/funder, who has granted medRxiv a license to display the preprint in perpetuity.
It is made available under a CC-BY 4.0 International license .

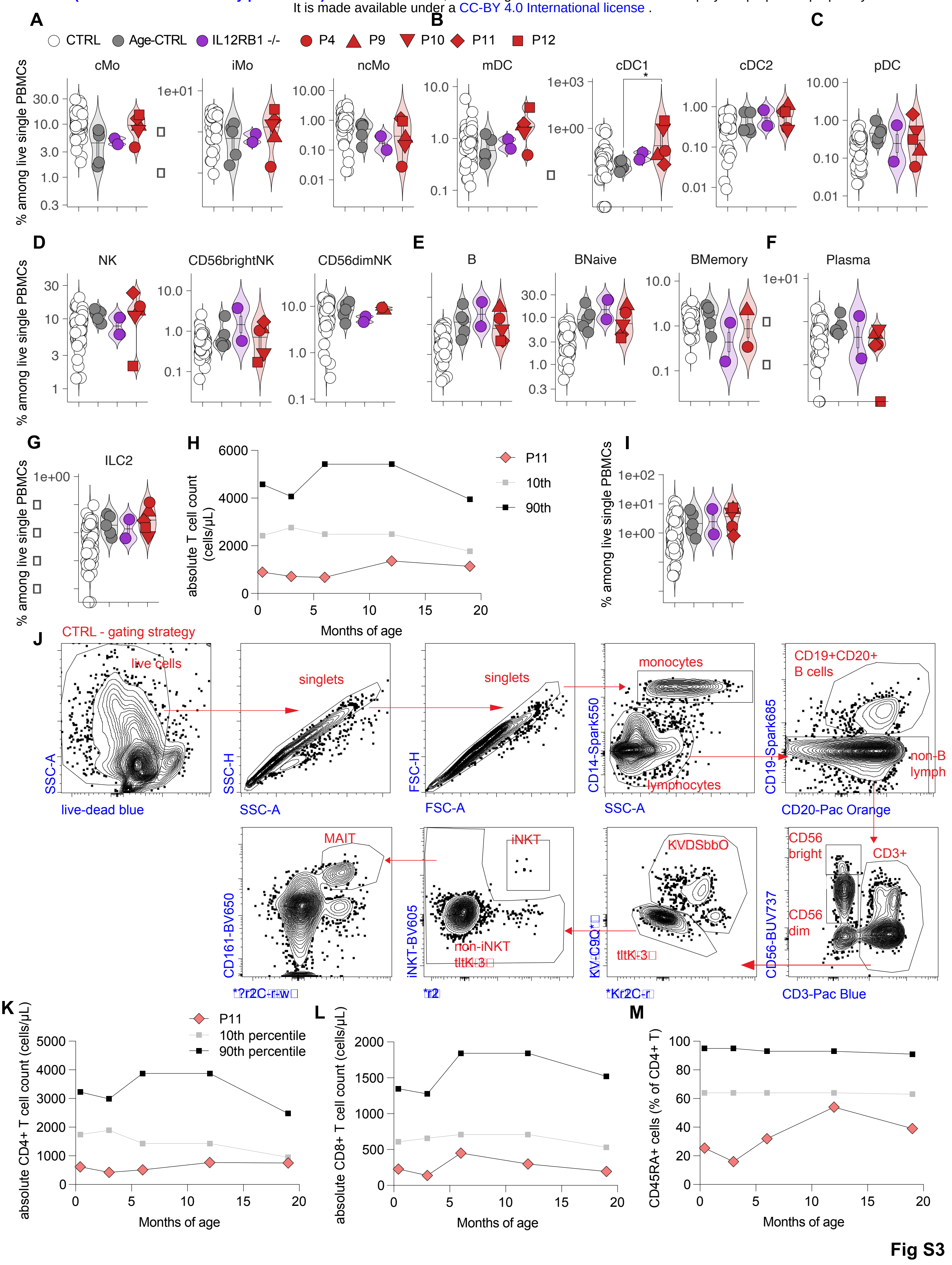

Fig S3

medRxiv preprint doi: https://doi.org/10.64898/2026.07.18.26358075; this version posted July 20, 2026. The copyright holder for this preprint (which was not certified by peer review) is the author/funder, who has granted medRxiv a license to display the preprint in perpetuity.
It is made available under a CC-BY 4.0 International license .

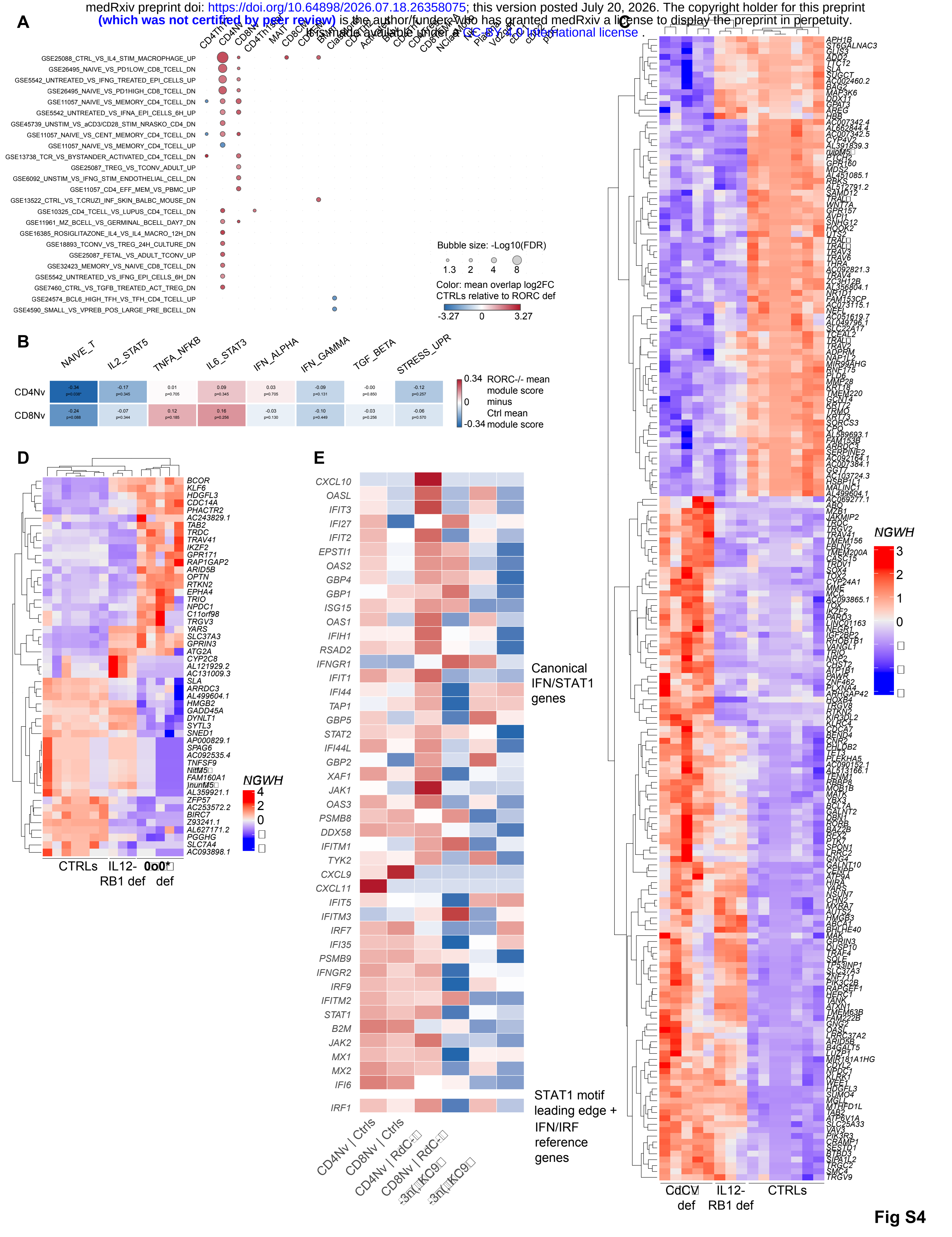

medRxiv preprint doi: https://doi.org/10.64898/2026.07.18.26358075; this version posted July 20, 2026. The copyright holder for this preprint (which was not certified by peer review) is the author/funder, who has granted medRxiv a license to display the preprint in perpetuity.
It is made available under a CC-BY 4.0 International license .

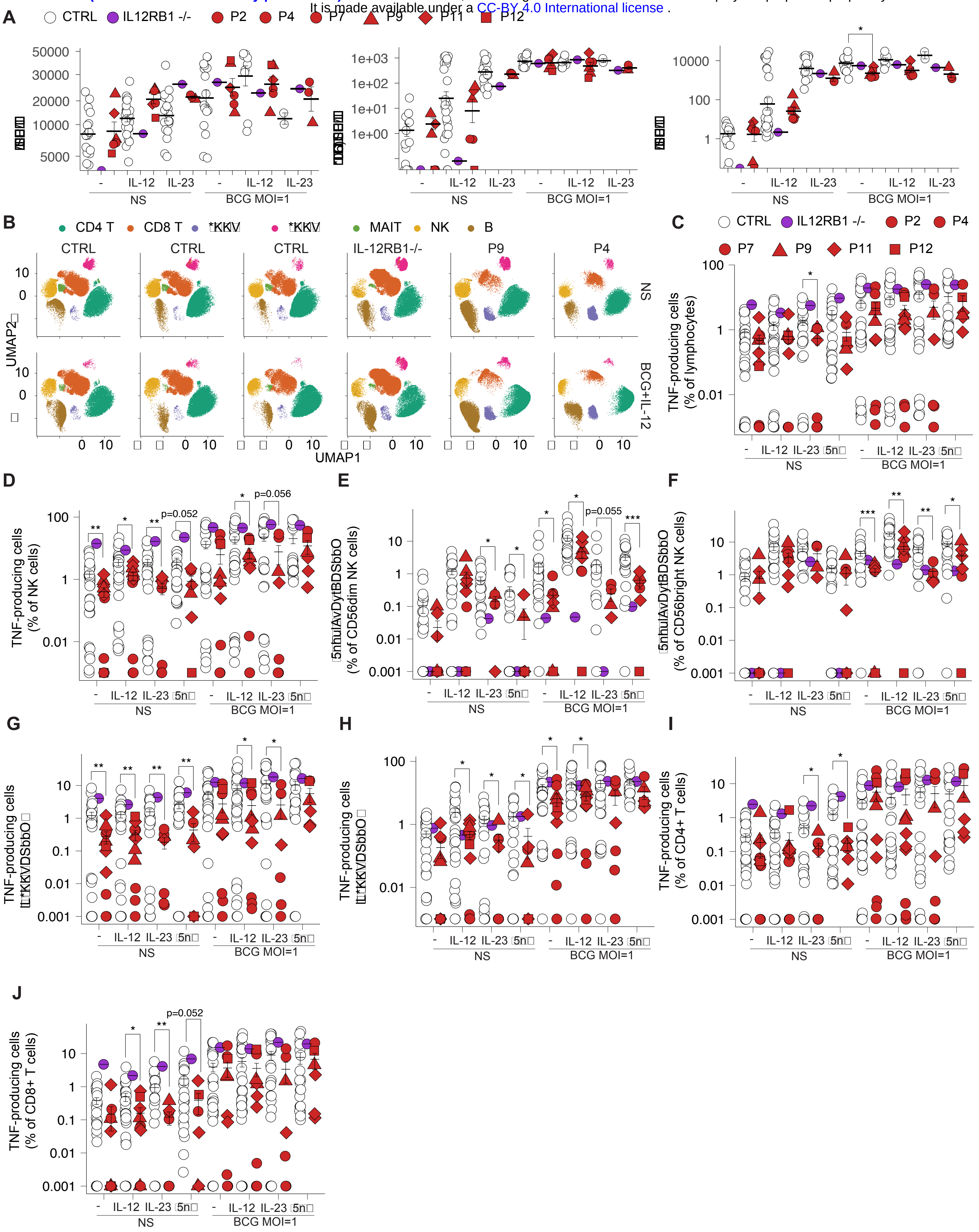

Fig S5

SYMBOL	CD4Nv_log2I	CD4Nv_AdjP	CD4Th1_log2	CD4Th1_Adj	CD4Th2_log2	CD4Th2_Adj	CD4Th17_lo	CD4Th17_Ad	CD4Th1star_	CD4Th1star_	CD4Treg_log	CD4Treg_Ad	CD8Nv_log2I	CD8Nv_AdjP	CD8CM_log2	CD8CM_AdjP	CD8EM_log2	CD8EM_AdjP	CD8TEMRA_	CD8TEMRA_	MAIT_log2F	MAIT_AdjP	Vd2gdT_log2	Vd2gdT_AdjP
ANLN	-0.0035855	NA	9.30E-09	NA	-5.52E-08	0.999978	NA	NA	NA	NA	-1.10E-07	0.99987372	-8.84E-04	0.99206849	7.91E-08	NA	2.13E-05	NA	-1.70E-07	0.99994558	-7.57E-04	NA	-3.40E-08	0.9999789
ARID5B	1.72814848	0.00150062	0.89561218	0.23889819	0.65092422	0.39367949	1.06513948	0.08578674	1.52804369	1.98E-04	2.58E-06	0.99987372	2.70530309	1.36E-05	1.42558798	0.0377047	0.01218223	0.66943007	3.24E-06	0.99994558	0.13714531	0.25901221	5.11E-06	0.9999789
ARRDC3	-1.0850796	0.00826262	-0.84844	0.18418033	-5.42E-06	0.999978	-0.045177	0.89083642	-0.0886988	0.97260302	-0.5344686	0.71448484	-1.3317616	0.0064266	-7.99E-06	0.63583568	-0.0072852	0.99968493	-3.72E-06	0.99994558	-0.0919379	0.71241345	2.18E-08	0.9999789
ATP6V1A	1.03428995	0.00619938	4.19E-06	0.99999693	5.52E-06	0.999978	0.00182386	NA	0.01398638	0.99925116	4.20E-06	0.99987372	0.04591458	0.7426645	4.29E-06	0.82006217	0.00858901	0.99968493	3.75E-06	0.99994558	-0.0068947	NA	4.73E-06	0.9999789
ATXN1	1.25311437	0.00720529	7.40E-06	0.74086231	1.08E-06	0.999978	0.00507411	0.99962555	0.05161353	0.99925116	2.70E-06	0.99987372	0.01521006	0.99206849	3.16E-06	0.99942098	0.0016383	0.99968493	4.99E-06	0.99994558	0.16083953	0.50441794	1.17E-06	0.9999789
AVPI1	-1.9709798	0.00267644	-1.5938868	0.0922103	-4.04E-06	0.85596215	-0.0080139	NA	-0.0216535	0.99925116	-1.79E-06	0.99987372	-0.0279923	0.99206849	-5.84E-06	0.10826112	-0.0088015	0.20267755	-2.13E-06	0.99994558	-0.0203518	NA	-4.08E-06	0.9999789
B4GALNT3	-0.0079977	NA	-1.29E-07	NA	-5.53E-08	0.999978	3.06E-04	NA	-0.0016791	6.61E-04	-7.56E-09	0.99987372	-4.13E-04	NA	4.75E-08	NA	-0.0019203	0.99968493	3.04E-07	0.99994558	0.00594074	NA	1.81E-07	0.9999789
BCL7A	1.34784876	0.00293873	2.78E-06	0.99999693	1.93E-06	0.999978	0.0038215	NA	0.00864111	0.99925116	1.47E-06	0.99987372	0.037066	0.99206849	3.12E-06	0.88477971	0.0041299	0.99968493	5.29E-07	0.99994558	-0.0029428	NA	-6.25E-08	0.9999789
BEND4	4.27364778	0.00770364	2.80E-07	NA	6.20E-07	0.999978	NA	NA	5.76E-04	0.99925116	-1.93E-08	0.99987372	7.08E-05	0.99206849	1.08E-06	NA	0.0016642	NA	9.12E-08	0.99994558	NA	NA	5.75E-08	0.9999789
BHLHE40	1.64759271	0.00266607	1.82436617	0.09384276	4.10E-06	0.999978	-0.0085675	0.99962555	0.00482692	0.99925116	4.11E-06	0.73674749	-0.001878	0.99636095	-4.50E-07	0.99942098	-0.0029342	0.99968493	-1.30E-06	0.99994558	0.02484365	0.94214259	-2.11E-06	0.9999789
CCDC110	-0.0466381	0.52447912	-1.03E-07	NA	-1.46E-06	0.00310378	NA	NA	2.01E-04	0.99925116	NA	NA	-9.97E-04	0.99206849	-1.51E-07	NA	1.80E-04	NA	NA	NA	-2.08E-05	NA	NA	NA
CCR6	-0.0349228	0.8444221	-5.37E-06	0.20041975	-6.21E-06	0.27178954	-0.0444327	0.03860575	-0.0048167	0.99925116	-2.5798559	0.00151915	-0.0012315	0.99206849	-1.52E-06	NA	-0.0043101	0.99968493	-8.50E-08	0.99994558	-2.8111967	0.09835299	-3.64E-07	0.9999789
CCR7	-0.0837876	0.75191893	-1.41E-06	0.99999693	-2.24E-06	0.999978	-1.9205548	0.00910528	0.01019191	0.99925116	1.37E-06	0.99987372	-0.0152376	0.99206849	-2.54E-06	0.99942098	-0.0032994	0.99968493	-3.23E-08	0.99994558	-0.0317464	NA	-1.80E-06	0.9999789
CCSER1	-0.0172512	0.94114288	-2.2292616	0.25736299	-3.69E-06	0.83523598	0.00278256	NA	-0.001462	0.99925116	2.69E-08	0.99987372	-7.47E-04	0.99206849	-2.46E-07	NA	-1.03E-04	NA	NA	NA	-0.0011598	NA	5.04E-08	0.9999789
CD8A	-0.0318888	0.91413166	-1.8334569	0.09849102	-2.4367117	0.05410372	-0.0010787	NA	0.00254592	0.99925116	-2.51E-06	0.99987372	-0.0579969	0.96527277	-1.9464116	0.02747759	-1.6657536	8.17E-06	-4.55E-06	0.99994558	-6.6474237	0.00632339	-1.72E-06	0.9999789
CD8B	-0.0646443	0.75851256	-2.3925864	0.00496559	-3.0646616	8.46E-05	-5.68E-04	NA	-0.0099125	0.99925116	-1.62E-06	0.99987372	-0.0772182	0.46720184	-2.7423933	5.86E-05	-2.4238788	9.86E-05	-4.87E-06	0.41413939	-0.0999402	NA	-4.11E-06	0.26637069
CDC14A	0.82793144	0.08382795	4.01E-06	0.99999693	6.09E-06	0.999978	0.02329559	0.98992441	0.06665223	0.95321396	5.56E-06	0.99987372	1.38568836	0.00266257	0.90321481	0.1612199	0.01332533	0.64286501	0.70285747	0.99994558	0.05304588	0.8823324	3.63E-06	0.9999789
CHN1	-0.0437109	0.62756186	-2.1153332	0.0362489	-2.3282804	7.57E-04	-0.0106836	0.99962555	-0.0122923	0.99925116	-3.29E-06	0.30261701	-0.0131958	0.99206849	-2.81E-06	NA	-0.0032912	0.87892132	8.72E-07	0.99994558	-0.0054609	NA	-5.02E-08	0.9999789
CHST2	2.46134556	9.15E-05	1.77E-06	0.99999693	2.19E-06	0.999978	7.37E-04	NA	0.00141542	0.99925116	8.62E-07	0.99987372	0.01791063	0.99206849	3.54668911	0.06970755	0.00223887	0.99419306	1.43E-06	0.99994558	-0.0015029	NA	-4.89E-07	0.9999789
CYP2C8	0.00116559	NA	7.16E-08	NA	NA	NA	NA	NA	NA	NA	NA	NA	-3.02E-04	4.31E-06	NA	NA	NA	NA	NA	NA	NA	NA	NA	NA
CYP4F12	-0.0025636	NA	9.21E-08	NA	-1.45E-07	0.00523053	-3.52E-05	NA	-8.86E-05	0.99925116	NA	NA	NA	NA	-1.07E-07	NA	-9.10E-05	NA	NA	NA	-1.78E-05	NA	NA	NA
CYP4V2	-1.2704318	6.05E-04	-0.8677059	0.26525329	-4.06E-06	0.999978	-0.0178419	0.87379249	-0.0138314	0.99925116	-2.95E-06	0.99987372	-0.0517153	0.99206849	-3.57E-06	0.99942098	-0.0063267	0.93192703	-1.00E-06	0.99994558	-0.034402	NA	-8.69E-07	0.9999789
DLGAP2	0.00669803	NA	-2.80E-07	NA	-1.04E-07	0.999978	1.99E-04	NA	-3.59E-04	0.99925116	-7.62E-08	0.99987372	-5.64E-04	0.99206849	6.97E-08	NA	-7.20E-05	NA	NA	NA	-9.14E-04	NA	1.15E-07	0.9999789
DUSP1	-0.2662799	0.45777934	-1.07E-05	0.61437901	-0.697258	0.01773339	-0.0267715	0.99962555	-0.3950428	0.67555732	-0.7106508	0.22606164	-0.0350704	0.99206849	-1.48E-05	0.42574573	-0.9182387	0.01643855	-1.16E-05	0.99994558	-1.1702498	0.00913391	-0.9190284	2.05E-05
EIF3E	-0.1628888	0.62943062	-3.54E-06	0.99999693	-3.60E-06	0.999978	-0.0251539	0.99962555	-0.0656619	0.99925116	-7.88E-06	0.99987372	-0.1144042	0.99206849	-4.82E-06	0.99942098	-0.0098887	0.99968493	-1.01E-05	0.99994558	-1.3051865	0.00759411	-4.54E-06	0.9999789
EPHA4	0.05467488	0.7295378	7.91E-07	0.99999693	-1.53E-06	0.999978	-0.0508	0.79190024	0.02806934	0.99925116	9.61E-07	0.99987372	3.5332191	5.45E-05	1.0400975	0.15904242	0.0020864	0.99968493	-1.20E-06	0.99994558	0.03379478	0.90856203	-2.03E-06	0.9999789
FBXL13	-0.0176854	0.94761377	-1.34E-06	0.99999693	-2.99E-07	0.999978	-4.97E-04	NA	-0.0015948	3.46E-09	3.37E-07	0.99987372	-0.0139247	0.99206849	3.10E-07	NA	4.76E-04	NA	-1.68E-07	0.99994558	-0.006007	NA	-2.22E-07	0.9999789
FLNB	0.07607706	0.81833591	3.10E-06	0.99999693	1.33590078	0.00641554	0.01053746	0.99962555	0.03241602	0.99925116	3.10E-06	0.99987372	0.00628724	0.99216591	2.26E-06	0.99942098	0.00175263	0.99968493	6.02E-07	0.99994558	-0.0140608	NA	-2.38E-07	0.9999789
GAB2	1.68458984	0.07741753	1.49549158	0.34032723	6.94E-06	0.54286972	2.26289022	0.00682068	0.02235272	0.99531306	3.19E-06	0.99987372	0.00354366	0.99206849	2.75E-06	0.71817295	0.00282324	0.99968493	4.45E-07	0.99994558	0.00409704	NA	-2.17E-06	0.9999789
GADD45A	-0.140498	0.53851039	-3.25E-06	0.99999693	-1.32E-06	0.999978	-0.0169018	0.99962555	-0.0043161	0.99925116	-2.28E-06	0.99987372	-1.8628203	0.00174065	-1.86E-06	0.99942098	-0.0047335	0.99968493	-4.14E-06	0.99994558	0.02211968	0.95755216	-3.92E-06	0.9999789
GATA3	0.61017271	0.1198682	5.56E-06	0.96220971	6.65E-06	0.999978	1.20157734	0.0082637	0.02278415	0.99925116	2.39E-06	0.99987372	0.05774695	0.83430679	3.28E-06	0.99942098	0.0125879	0.85233749	3.36E-06	0.99994558	-0.113292	0.68184513	3.79E-06	0.9999789
GLIS3	-2.8479949	0.00823177	-3.52E-06	0.36642366	-3.49E-07	0.999978	-4.58E-05	NA	-0.0026309	0.99925116	-5.59E-07	0.99987372	-0.0349159	0.44537039	-3.06E-06	NA	-0.0028914	NA	NA	NA	-0.0024223	NA	-7.87E-08	0.9999789
GPR135	-0.0514757	0.69576407	1.31E-07	0.99999693	6.00E-07	0.999978	-0.0025622	NA	-0.0055929	0.99925116	2.98E-07	0.99987372	-0.0047205	0.99879076	-6.40E-07	NA	-5.91E-04	0.99968493	3.24E-07	0.99994558	-0.0061791	NA	-1.05E-06	0.9999789
GPRIN3	1.50210249	0.0494502	4.48E-06	0.99999693	3.43E-06	0.999978	-0.9038662	0.20220708	0.07243235	0.99925116	4.38E-06	0.99987372	2.31606134	4.21E-04	4.75E-06	0.94136432	0.0195137	0.50838121	4.94E-06	0.99994558	0.02884826	0.95478781	2.50E-06	0.9999789
HDGFL3	3.51242426	9.93E-21	2.73346658	0.00175305	1.87755972	0.0076519	0.00817557	0.89083642	2.38558714	0.05041841	1.69E-06	0.99987372	3.81331346	1.34E-06	1.65890217	0.06970755	0.01276934	0.58135477	3.19E-07	0.99994558	0.07929896	NA	-3.63E-06	0.9999789
HERC1	1.1130396	0.0078164	7.81E-06	0.42698493	7.20E-06	0.999978	0.06444553	0.38195936	0.8849238	0.46309539	0.69130746	0.32933761	0.47950169	0.33616909	1.10173352	0.02747759	0.62632134	0.28697396	6.56E-06	0.99994558	0.71356217	0.19033445	8.13E-06	0.9999789
HOXB4	2.09928353	0.00817044	3.15E-06	0.99008192	2.86E-06	0.999978	0.00304586	NA	0.01401493	0.99925116	2.14E-06	0.99987372	0.01494026	0.95375244	1.50E-06	NA	0.00625896	0.86728464	2.21E-06	0.99994558	0.01677386	NA	3.79E-06	0.9999789
HSBP1L1	-1.0721235	0.00377575	-0.5716158	0.37036269	-3.30E-06	0.999978	-0.0167421	0.9124532	-0.0307299	0.99925116	-1.95E-06	0.99987372	-0.0500869	0.99206849	-2.38E-06	0.99942098	-0.0032769	0.99968493	-6.00E-07	0.99994558	-0.0409375	NA	-2.51E-06	0.9999789
ID2	-0.0559504	0.88847256	8.66E-08	0.99999693	-4.74E-06	0.999978	-0.0210376	0.99962555	-1.1175823	0.00511901	1.56E-06	0.99987372	-0.0435057	0.99206849	-3.18E-06	0.99942098	-0.0068883	0.99968493	-1.67E-06	0.99994558	-0.0427038	0.80615434	-2.93E-06	0.9999789
IGF2BP2	3.08640181	0.0015963	-3.68E-07	0.99999693	2.30E-07	0.999978	NA	NA	8.27E-05	0.99925116	-2.48E-08	0.99987372	-8.34E-04	0.99206849	1.01E-06	NA	5.88E-04	NA	2.42E-07	0.99994558	-0.0010614	NA	8.01E-09	0.9999789
IGF2BP3	0.01765555	NA	8.01E-07	0.99999693	1.48E-07	0.999978	4.41E-04	NA	0.00144718	0.99925116	-4.40E-08	0.99987372	0.0036802	0.93362999	-1.22E-07	NA	-1.30E-04	NA	4.41E-08	0.99994558	-2.96E-05	NA	1.09E-07	0.9999789
IGFBP3	-0.0417961	0.22327287	-2.47E-06	0.13353531	-3.39E-06	0.09913219	-2.06E-04	NA	-0.0436964	0.06695145	-3.87E-08	0.99987372	-0.0080818	0.99237055	-2.85E-06	NA	-3.2061375	0.00310548	-1.00E-06	0.99994558	-0.0104548	NA	-2.92E-06	0.8926471
IKZF2	2.54210837	3.47E-05	3.90E-06	0.7823334	3.38E-06	0.999978	-4.29E-04	NA	0.00183628	0.99925116	-7.03E-07	0.99987372	1.97352961	0.03718826	1.94140194	0.02692382	0.00809848	0.29607928	-1.98E-06	0.99994558	-0.0807225	0.51721784	1.20E-06	0.9999789
IL3	NA	NA	NA	NA	6.68E-08	0.999978	NA	NA	NA	NA	NA	NA	NA	NA	NA	NA	NA	NA	NA	NA	NA	NA	NA	NA
JAKMIP2	2.86008542	1.41E-05	-4.91E-07	0.99999693	-1.00E-06	0.999978	0.00715061	NA	0.0071068	0.99925116	1.09E-06	0.99987372	-8.41E-04	0.99214083	1.94E-06	0.99942098	0.00155113	0.99968493	-1.99E-06	0.99994558	0.01824257	NA	5.85E-07	0.9999789
KIR3DL2	4.0288579	5.26E-04	3.076162	0.09384276	3.01152137	0.05410372	7.45E-04	NA	0.00441706	0.99925116	3.86E-07	0.99987372	9.52E-04	0.99206849	1.89153576	NA	8.26E-04	0.99968493	-1.36E-06	0.99994558	-9.42E-04	NA	-6.38E-07	0.9999789
KLF6	1.19065795	0.07394822	0.8637432	0.39348252	5.94E-06	0.999978	0.0213392	0.96863741	0.03292109	0.99925116	6.58E-06	0.99987372	1.94997327	4.78E-04	0.76114767	0.37047746	0.00877806	0.99968493	-1.75E-06	0.99994558	0.17598203	0.47634871	3.70E-06	0.9999789
KLRF1	-0.0396761	0.83704577	-5.13E-07	NA	-5.83E-07	0.999978	3.72E-04	NA	-0.0043419	0.99925116	-4.08E-07	0.99987372	-0.0018713	0.9944897	-4.48E-07	NA	-0.0094169	0.49278822	-1.8702128	5.98E-04	-0.0983105	NA	-1.937571	0.21921838
KRT18	-2.4685649	0.00196441	-4.23E-06	0.61027843	-2.18E-06	0.999978	-0.0011528	NA	-0.0095831	0.99925116	-1.14E-06	0.99987372	-0.0071769	0.99206849	-2.16E-06	NA	-0.0030433	NA	NA	NA	-0.00532	NA	-5.14E-08	0.9999789
KRT72	-2.0269292	0.00698792	-1.70E-06	0.99999693	-2.61E-07	0.999978	NA	NA	-0.0013613	0.99925116	1.10E-07	0.99987372	-0.0123524	0.99206849	-4.43E-07	NA	-0.0014223	NA	-1.56E-07	0.99994558	-0.0014776	NA	NA	NA
LGALS1	-0.0562052	0.86872436	-7.41E-06	0.8657606	-0.9475764	0.00126228	-0.0125183	0.99962555	-0.0111539	0.99925116	-7.01E-07	0.99987372	0.0087241	0.99206849	-2.78E-06	0.99942098	2.72E-04	0.99968493	-1.03E-06	0.99994558	0.02077902	0.94214259	-1.62E-07	0.9999789
LGALS3	0.00746605	0.98718482	2.97E-07	0.99999693	-1.11E-05	0.06682955	-2.5583611	0.00398644	-0.0509816	0.82509468	-3.05E-06	0.99987372	-0.0056448	0.99206849	-3.93E-06	0.95532801	-0.0113335	0.75983095	-3.18E-06	0.99994558	-2.9376923	0.12591901	-2.75E-06	0.9999789
LIN7A	-0.0140062	NA	-7.59E-08	NA	1.30E-07	0.999978	NA	NA	NA	NA	5.83E-08	0.99987372	-8.46E-04	0.99206849	NA	NA	-1.15E-04	NA	NA	NA	-4.58E-04	NA	NA	NA
LYAR	0.08909695	0.71477096	2.14E-06	0.99999693	-7.57E-08	0.999978	-1.9521671	0.0029405	-0.3743462	0.77341561	-6.11E-07	0.99987372	0.04659186	0.99206849	-4.63E-07	0.99942098	-0.0053833	0.99968493	-2.57E-06	0.99994558	-0.0647776	0.84881314	-2.92E-06	0.9999789
LYZ	-1.1822637	0.05520251	-9.38E-06	0.12244283	-7.62E-06	0.14520406	-0.0314801	0.82297538	-0.0500978	0.77341561	-1.06E-05	0.0470469	-0.0769889	0.55902988	-1.2983131	0.11195122	-1.399247	0.00222172	-5.65E-06	0.99994558	-0.0942182	NA	-6.15E-06	0.9999789
MGAT4A	-0.5468477	0.12210959	-7.82E-06	0.88497672	-1.46E-05	0.83523598	-1.5033807	0.0026722	-0.4557955	0.7289239	-7.86E-06	0.99987372	-0.529701	0.43247145	-1.30E-05	0.18987412	-0.0180616	0.66862089	-2.95E-06	0.99994558	-1.0676085	0.09650661	-5.37E-06	0.9999789
MME	6.41930113	3.35E-11	3.31831239	0.19762333	1.91E-06	0.39367949	7.68E-04	NA	5.19E-04	0.99925116	-2.81E-07	0.99987372	0.00127667	0.99206849	1.61E-06	0.51777497	0.00156895	0.3382127	1.21E-07	0.99994558	NA	NA	NA	NA
MMP28	-3.149313	8.34E-06	-2.42E-06	0.3101985	-1.33E-07	0.999978	-3.97E-05	NA	-4.62E-04	0.99925116	-2.88E-06	0.99987372	-0.0409235	0.42895931	-1.36E-06	NA	-0.0016839	NA	1.67E-08	0.99994558	-0.0013152	NA	NA	NA
MOB1B	1.01016457	0.00293873	3.13E-06	0.99999693	4.73E-06	0.999978	9.28E-05	0.99962555	0.0146221	0.99925116	2.08E-06	0.99987372	0.02422069	0.99206849	5.10E-06	0.62376025	0.01485159	0.50838121	2.43E-06	0.99994558	0.01908192	NA	1.48E-06	0.9999789
MPDZ	NA	NA	NA	NA	NA	NA	NA	NA	NA	NA	NA	NA	NA	NA	NA	NA	NA	NA	NA	NA	NA	NA	NA	NA
MYC	0.04178144	0.89456501	4.39E-06	0.99999693	6.37E-06	0.999978	1.60102137	0.00910528	0.02543065	0.99925116	-7.67E-07	0.99987372	0.02782469	0.99206849	1.57E-06	0.99942098	0.00724038	0.97156544	5.02E-08	0.99994558	0.01415042	0.96005986	3.78E-06	0.9999789
MZB1	2.8425997	0.00191466	1.16E-06	0.99999693	7.20E-07	0.999978	-5.34E-04	NA	0.00324984	0.99925116	2.12E-07	0.99987372	0.01224056	0.99206849	1.36E-06	NA	0.00118918	0.99968493	7.10E-07	0.99994558	-0.0167096	NA	9.07E-07	0.9999789
NCL	-0.2420552	0.48973934	-7.53E-06	0.99999693	-4.50E-06	0.999978	-0.0335097	0.99962555	-0.0922803	0.99925116	-6.08E-06	0.99987372	-0.453826	0.57688655	-6.29E-06	0.99942098	-0.008142	0.99968493	-7.79E-06	0.99994558	-0.8330412	0.00913391	-7.83E-06	0.9999789
NDFIP1	-0.5589141	0.10802296	-1.29E-05	0.90112497	-6.23E-06	0.999978	-0.0342101	0.99962555	-0.0291609	0.99925116	-3.94E-06	0.99987372	-0.8763746	0.0029582	-9.34E-06	0.71817295	-0.0125966	0.99968493	-2.51E-06	0.99994558	0.03746097	0.92546623	5.02E-08	0.9999789
NEFL	-5.9273178	9.47E-04	-4.58E-06	0.09384276	-7.35E-06	0.24085028	-0.0089919	0.99962555	-0.0041041	0.99925116	NA	NA	NA	NA	NA	NA	-0.0011672	NA	NA	NA	-0.0011754	NA	NA	NA
NEGR1	3.29952303	0.00588145	1.97E-07	NA	1.60E-07	0.999978	5.16E-04	NA	9.88E-05	0.99925116	5.25E-08	0.99987372	-6.20E-04	0.99206849	2.62E-07	NA	4.30E-04	NA	NA	NA	-8.58E-04	NA	-6.74E-08	0.9999789
NPDC1	2.58485289	5.26E-04	1.16157825	0.12244283	7.59E-06	0.51091965	0.06405547	0.39279686	0.02416751	0.99925116	3.21E-06	0.99987372	3.31397861	2.45E-06	1.21E-06	0.99942098	0.00237756	0.99968493	-2.28E-08	0.99994558	0.07699864	NA	2.37E-06	0.9999789
NR3C2	-0.0550045	0.74854754	-1.38E-06	0.99999693	-3.41E-06	0.999978	-4.3605618	0.00682068	-0.0123765	0.99925116	-1.52E-06	0.99987372	-0.0162854	0.99206849	-2.25E-06	0.99942098	-1.4360974	0.22806138	-2.3693776	0.3489762	-0.1212142	NA	-2.75E-06	0.9999789
NRP2	3.12018644	0.00823177	1.36E-06	0.71464483	9.98E-07	0.999978	9.01E-04	NA	6.42E-04	0.99925116	6.28E-07	0.99987372	-3.70E-04	0.99206849	1.58E-07	NA	9.29E-04	NA	NA	NA	NA	NA	1.48E-07	0.9999789
NTRK2	-1.73E-04	NA	8.21E-09	NA	-1.05E-06	0.999978	-0.0012819	NA	1.09E-04	0.99925116	-1.98E-07	1.45E-11	NA	NA	-9.91E-08	NA	-5.11E-05	NA	NA	NA	-4.78E-04	NA	5.58E-08	0.9999789
PARD3	2.60806454	2.93E-04	8.41E-07	0.99999693	9.96E-07	0.999978	4.78E-04	NA	0.00291626	0.99925116	5.47E-08	0.99987372	0.00187	0.99206849	1.73E-06	NA	0.00152145	0.66862089	3.87E-07	0.99994558	-4.66E-04	NA	2.00E-08	0.9999789
PCBP3	0.16167656	0.31422839	4.49E-06	0.71516237	3.80E-06	0.89754547	7.59E-04	0.99962555	0.00974207	0.99925116	3.47E-06	0.8042988	0.00725444	0.99206849	3.00E-06	0.3796034	3.61E-04	0.99968493	-9.01E-09	0.99994558	-0.0087709	NA	-9.25E-09	0.9999789
PDLIM5	0.11045807	0.67642517	7.36E-07	0.99999693	5.08E-06	0.97912926	2.99029319	0.00421392	0.04448682	0.97260302	6.34E-07	0.99987372	-0.0035214	0.99334835	8.61E-07	0.99942098	0.00127542	0.99968493	1.15E-06	0.99994558	-0.0220858	NA	6.18E-09	0.9999789
PHLDA1	0.0650702	0.61806061	2.65E-06	0.99999693	2.42E-06	0.999978	1.93321909	0.00682068	0.00957054	0.99925116	1.95E-06	0.99987372	3.77E-04	0.99214083	2.84E-07	0.99942098	-0.0014395	0.99968493	4.93E-07	0.99994558	-0.0346376	NA	-4.89E-06	0.9999789
PHLDB2	2.87412937	3.47E-04	2.18E-06	0.94448946	1.69E-06	0.999978	-0.0022798	0.99962555	0.02227319	0.99925116	3.14E-08	0.99987372	0.01313539	0.99206849	2.74E-06	0.43076957	0.00199545	0.99968493	2.58E-06	0.99994558	0.02366334	NA	1.80E-06	0.9999789
PI16	-0.0407953	0.55267255	-2.02E-06	0.73572036	-3.0437969	0.00132409	-0.0045375	NA	-0.0011347	0.99925116	-3.53E-06	0.87917422	-0.0014642	0.99636095	2.22E-07	NA	-4.45E-04	NA	NA	NA	-0.0018687	NA	-1.99E-07	0.9999789
PIK3C2B	1.90434674	0.00723756	4.05E-06	0.66168953	4.27E-06	0.999978	0.02768586	0.82297538	0.01355249	0.99925116	4.11E-06	0.99987372	0.02228679	0.99206849	2.52E-06	0.99942098	0.00479195	0.99968493	1.29E-06	0.99994558	0.03623954	NA	1.28E-06	0.9999789
PLAAT3	0.10457601	0.61084092	2.78E-07	0.99999693	0.901849	0.2086256	2.27654616	0.00879157	0.0115482	0.99925116	2.42E-06	0.99987372	9.74E-04	0.99211075	2.88E-07	0.99942098	0.00303871	0.99968493	1.16E-06	0.99994558	0.00136529	NA	3.03E-06	0.9999789
PLCL1	-0.0838938	0.72830284	-3.46E-06	0.99999693	-6.02E-06	0.999978	-0.0498201	0.52808853	-0.0098134	0.99925116	-8.78E-06	0.81753212	-0.0197081	0.99206849	-2.91E-06	0.99942098	-0.0043312	0.92815791	-4.0165625	5.98E-04	-0.0226572	NA	-3.75E-06	0.44141487
PLD6	-1.5973961	0.00230464	-4.05E-06	0.88412452	-3.14E-06	0.999978	-0.0039243	NA	-0.0077477	0.99925116	-2.11E-06	0.99987372	-0.029306	0.99206849	-1.59E-06	NA	-0.0024936	0.99968493	-1.96E-07	0.99994558	-0.001215	NA	-9.97E-07	0.9999789
PRICKLE2	0.01165643	NA	-6.75E-07	NA	4.24E-08	0.999978	NA	NA	NA	NA	NA	NA	-1.20E-05	NA	NA	NA	-8.16E-05	NA	-9.69E-08	0.00317697	NA	NA	NA	NA
PTCH2	-1.4644856	0.00887869	-5.44E-06	0.42666926	-2.90E-06	0.999978	-0.0077538	0.99962555	-0.0178972	0.97260302	-1.07E-06	0.99987372	-0.0181213	0.78633977	-4.85E-06	0.15241038	-0.0081237	0.66862089	-5.79E-07	0.99994558	-0.1462197	NA	-2.80E-06	0.9999789
PTPN13	-7.34E-04	0.99883798	-7.18E-07	0.99999693	-1.72E-06	0.999978	-3.7619062	0.0029405	-0.011005	0.99925116	1.06E-06	0.99987372	NA	NA	6.51E-08	NA	1.74E-04	NA	NA	NA	-0.0168038	NA	-2.28E-08	0.9999789
RADX	-0.1141879	0.519909	-3.57E-06	0.99008192	-2.4327606	3.01E-05	-0.0249987	0.39279686	-0.0395916	0.66384102	-2.89E-06	0.99987372	-0.0065447	0.99206849	-2.58E-06	0.99942098	-0.0094267	0.29607928	-1.76E-06	0.99994558	-0.0482092	NA	-3.5067692	0.0025954
RAPGEF1	1.31290963	0.00252578	8.23E-06	0.42698493	3.75E-06	0.999978	-0.0267968	0.99962555	0.56315943	0.50986109	4.01E-06	0.99987372	0.07214961	0.57688655	6.94E-06	0.42572497	0.01064775	0.99968493	1.53E-06	0.99994558	0.68198789	0.20780238	4.69E-06	0.9999789
RBBP8	1.79214645	0.00112031	1.30603071	0.19762333	3.95E-06	0.94672103	0.00273752	0.99962555	0.0032432	0.99925116	2.29E-06	0.99987372	0.00956597	0.99206849	3.49E-06	0.54133676	0.00479666	0.96678934	1.65E-06	0.99994558	0.01149627	NA	5.05E-07	0.9999789
RFX2	2.11702516	8.50E-05	1.27792846	0.19762333	3.37E-06	0.999978	0.02466682	0.9124532	1.75435321	0.15111988	1.96E-06	0.99987372	0.04693875	0.99206849	1.7177087	0.16991399	0.00472021	0.82705106	7.81E-08	0.99994558	-0.0133819	NA	3.71E-07	0.9999789
RIC3	-0.8016681	0.16295056	-9.98E-06	0.26590925	-7.10E-06	0.90603828	-0.0266212	0.89214193	-0.0083394	0.99925116	-2.37E-06	0.99987372	-1.1018187	0.33616909	-4.75E-06	0.7721256	-1.7903067	0.00222172	-2.2194682	0.93883455	-0.0150406	NA	-4.81E-06	0.9999789
RNF175	-3.4953584	2.35E-04	-3.23E-06	0.21331545	-6.29E-08	0.999978	NA	NA	NA	NA	-6.79E-07	0.99987372	-0.0038544	0.99935073	-9.64E-08	NA	-5.50E-04	NA	NA	NA	NA	NA	NA	NA
RORB	6.21989512	7.14E-06	1.40E-06	0.44140935	5.70E-07	0.999978	-4.46E-05	NA	0.00123692	0.99925116	4.75E-08	0.99987372	-1.82E-04	0.99206849	1.38E-06	NA	9.60E-04	0.83556763	3.45E-07	0.99994558	-2.89E-05	NA	3.50E-08	0.9999789
RTKN2	4.67678134	1.96E-65	3.43471277	1.03E-22	2.92212194	1.47E-11	3.25839616	0.0122461	4.47702129	1.25E-08	1.12E-05	0.92360256	4.84358193	1.34E-06	2.4533332	1.65E-04	3.63226628	3.94E-09	4.97E-07	0.99994558	0.14784145	NA	5.06E-07	0.9999789
S100B	0.05531668	0.55267255	1.01E-06	0.99999693	-6.04E-07	0.999978	-5.82E-04	NA	-0.0010256	0.99925116	-6.03E-07	6.13E-07	-0.0064397	0.99206849	-1.11E-06	0.99942098	-0.0018381	0.99968493	-1.13E-07	0.99994558	-0.0178946	NA	-6.19E-07	0.9999789
SAMD12	-0.1676401	0.02250018	-3.33E-06	0.20081646	-5.48E-06	0.01545728	-0.0360353	0.02463375	-0.0072565	0.99925116	-2.71E-06	0.8105428	-0.0746718	0.14778156	-2.52E-06	0.49343	-0.0065053	0.08191078	-1.39E-07	0.99994558	-0.0565852	NA	-3.05E-06	0.71879787
SESTD1	1.77089123	2.26E-04	3.47E-06	0.98797918	1.72E-06	0.999978	-7.53E-05	NA	0.00419818	0.99925116	1.32E-06	0.99987372	2.01640795	0.31682709	3.11E-06	0.84721605	0.00340914	0.99968493	-1.66E-06	0.99994558	-7.63E-04	NA	7.47E-08	0.9999789
SIPA1L2	2.9003211	0.00185955	1.30E-06	0.99999693	3.98E-06	0.58959497	4.00E-04	NA	0.00427532	0.99925116	1.47E-06	0.99987372	0.00126008	0.99206849	1.65E-06	NA	0.00247011	0.99968493	-4.39E-08	0.99994558	0.01475648	NA	-1.60E-06	0.9999789
SLC22A17	-2.2386495	0.00549697	-3.94E-06	0.3101985	-1.95E-06	0.999978	-5.55E-04	NA	-0.0013534	0.99925116	-4.19E-08	0.99987372	-0.0081577	0.99206849	-1.44E-06	NA	-0.001674	NA	NA	NA	-0.0010326	NA	-3.25E-07	0.9999789
SLC37A3	1.28323309	0.03368438	1.15312589	0.18418033	5.37E-06	0.85596215	0.01400569	0.87514444	0.03462299	0.95321396	3.00E-06	0.99987372	2.010823	0.00266257	2.67E-06	0.99942098	0.00518985	0.99419306	1.29E-06	0.99994558	0.14073818	0.19471064	9.91E-07	0.9999789
SLC7A4	NA	NA	NA	NA	NA	NA	NA	NA	NA	NA	NA	NA	-7.99E-04	1.36E-05	NA	NA	NA	NA	NA	NA	NA	NA	NA	NA
SMC4	1.40522727	0.00293873	6.50E-06	0.95339577	2.67E-06	0.999978	0.0557973	0.27302602	0.03259034	0.99925116	1.80E-06	0.99987372	0.06773637	0.36665817	1.62158613	0.02046043	0.00829426	0.83556763	-1.17E-07	0.99994558	0.0023446	0.99436378	6.00E-06	0.9999789
SNED1	-0.0331414	0.94645109	1.39E-06	0.99999693	-1.07E-06	0.999978	0.01029037	0.84359047	0.01550332	0.99925116	-8.86E-07	0.99987372	-3.146333	0.00266257	-5.38E-06	0.84735344	0.00171892	0.99968493	-4.28E-07	0.99994558	0.00635141	NA	2.58E-07	0.9999789
SNRK	-0.0817511	0.83685617	-5.63E-06	0.99999693	-7.18E-06	0.999978	-1.3144571	0.00879157	-0.0551269	0.99925116	-2.33E-06	0.99987372	-0.0862678	0.99206849	-2.93E-06	0.99942098	-0.0070776	0.99968493	-7.30E-06	0.99994558	-0.0548382	0.89919259	-5.41E-06	0.9999789
SOX4	2.00293471	0.00277052	1.32229489	0.19762333	2.11991151	0.0480127	4.78324394	0.01360121	3.24505133	0.0354997	1.17E-06	0.99987372	0.02922922	0.88582928	1.23E-06	0.99942098	0.00183456	0.95132465	4.31E-07	0.99994558	-0.0090577	NA	-8.03E-07	0.9999789
SUMO4	1.67352644	1.36E-05	0.94448966	0.21331545	9.21E-06	0.05598079	0.00993033	NA	0.01016213	0.99925116	3.12E-06	0.99987372	0.04060853	0.79149711	2.86E-06	0.7721256	0.00237296	0.99968493	7.68E-07	0.99994558	0.04817792	NA	4.65E-07	0.9999789
TAB2	1.02093894	0.00272261	0.69392256	0.19762333	0.79736495	0.01785267	0.02641407	0.9124532	0.09424742	0.99925116	0.77944462	0.06715108	1.07157017	0.03315484	7.45E-06	0.49478714	0.00644419	0.99968493	3.35E-06	0.99994558	0.0352492	0.9085186	2.64E-06	0.9999789
TBX3	0.00133952	NA	1.12E-07	NA	-4.05E-08	0.999978	NA	NA	NA	NA	NA	NA	NA	NA	NA	NA	2.03E-04	NA	NA	NA	NA	NA	6.84E-08	0.9999789
TCEAL2	-0.151098	0.0022402	-4.00E-06	0.02635415	-1.36E-06	0.999978	-0.0010167	NA	-5.77E-04	0.99925116	NA	NA	-0.0152185	0.99206849	-8.24E-07	NA	-0.0018321	NA	NA	NA	-4.99E-04	NA	-1.02E-07	0.9999789
TMEM220	-1.8637666	0.00230464	-3.53E-06	0.94102163	1.87E-07	0.999978	-3.14E-04	NA	-0.0049938	0.99925116	-2.84E-07	0.99987372	-0.0347798	0.99206849	-1.92E-06	NA	-0.0016883	NA	-7.86E-08	0.99994558	-0.0049918	NA	-2.78E-08	0.9999789
TNFSF10	0.27939879	0.23528235	4.82E-06	0.37036269	3.33E-06	0.999978	2.33029124	0.00398644	0.01986947	0.99925116	3.47E-06	0.99987372	0.01900372	0.99206849	1.48E-06	0.99942098	0.00890975	0.56553531	1.20E-06	0.99994558	0.04000033	NA	4.16E-06	0.9999789
TOX2	2.76105799	6.15E-06	4.24E-06	0.66978813	1.22656105	0.19493931	-2.71E-04	NA	0.00143671	0.99925116	1.36069236	0.19115671	0.00330016	0.99206849	4.29001056	0.01667633	0.00286746	0.67444298	5.47E-07	0.99994558	0.00548232	NA	4.99E-07	0.9999789
TP53INP1	2.34713329	2.93E-04	6.67E-06	0.54631395	5.08E-06	0.999978	0.0276573	0.84359047	0.02006254	0.99925116	3.62E-06	0.99987372	0.00624816	0.99206849	2.86E-06	0.99942098	0.8480795	0.40727453	6.29E-06	0.99994558	0.0160818	NA	1.72E-06	0.9999789
TRIO	2.93987022	1.88E-07	1.50E-06	0.99999693	1.00E-06	0.999978	-1.75E-04	NA	0.00558671	0.99925116	5.26E-07	0.99987372	2.85130539	0.0095084	3.71254963	2.75E-04	0.00375988	0.99968493	1.18E-06	0.99994558	-0.0015182	NA	1.03E-06	0.9999789
TRMO	-1.2626441	8.34E-05	-1.0361232	0.06105358	-9.66E-06	0.77036505	-0.029829	0.83474434	-0.0373255	0.97260302	-3.05E-06	0.99987372	-0.045915	0.99206849	-8.31E-06	0.20042531	-0.0165923	0.66862089	-2.85E-06	0.99994558	-0.0243057	NA	-1.71E-06	0.9999789
TSC22D3	-0.1290307	0.66365326	-4.35E-06	0.99999693	-1.01E-05	0.999978	-0.0227109	0.99253065	-0.0702381	0.99925116	-7.96E-06	0.99987372	-0.1003812	0.91459589	-9.24E-06	0.90569852	-0.0198395	0.58330219	-9.65E-06	0.99994558	-1.0226927	0.00913391	-0.4056361	0.93833454
TSHZ2	-0.065383	0.49133774	-1.44E-06	0.99999693	-3.07E-06	0.999978	-0.0010057	0.99962555	-0.0058196	0.99925116	4.15E-07	0.99987372	-0.0389565	0.4951396	-3.4631115	5.86E-05	-0.0014742	0.99968493	-6.09E-07	0.99994558	-0.0124227	NA	-5.27E-07	0.9999789
TTC12	-1.6977127	0.00588145	-7.79E-06	0.20081646	-1.82E-06	0.999978	-0.0121226	0.99962555	-0.0133976	0.99925116	-8.14E-07	0.99987372	-0.0420026	0.83430679	-3.13E-06	NA	-0.0034662	0.99968493	-8.52E-10	0.99994558	-0.0080185	NA	6.80E-07	0.9999789
UTS2	-0.0823425	0.02702651	-8.33E-07	0.72262567	-2.68E-06	0.16064147	-0.0013531	NA	-0.0020927	0.99925116	-3.49E-06	0.37055837	-0.0025006	0.0095084	-1.92E-06	NA	-0.0067609	0.42631984	-2.01E-06	0.99994558	-0.0120098	NA	-2.81E-06	0.9999789
VANGL1	4.30998816	3.18E-05	1.88E-06	0.99999693	1.39E-06	0.999978	5.98E-04	NA	0.00992339	0.99925116	7.42E-07	0.99987372	4.62E-04	0.99206849	6.95E-07	NA	0.00398853	0.99968493	-1.27E-06	0.99994558	-0.001587	NA	8.03E-07	0.9999789
VAV3	1.77719252	3.18E-05	5.24E-06	0.70545628	3.22E-06	0.999978	-0.0023207	NA	0.04533859	0.50986109	-2.78E-08	0.99987372	0.02778908	0.99206849	1.16541084	0.10826112	0.00373486	0.99968493	8.76E-07	0.99994558	0.09980542	0.34510967	2.31E-07	0.9999789
WNT7A	-1.4289574	0.00723756	-4.63E-06	0.36348538	-1.7878191	0.01428639	-0.0190192	0.6690752	-0.0184917	0.99925116	3.58E-08	0.99987372	-0.0111158	0.99206849	-3.43E-06	NA	-0.0039808	NA	-8.66E-08	0.99994558	-0.002503	NA	-3.66E-07	0.9999789
YBX3	2.28926345	1.93E-08	1.64716653	0.00333277	1.12430076	0.24671086	3.2482474	0.00398644	2.33329334	0.01661545	1.86827976	0.0470469	0.0668609	0.99206849	1.19E-06	0.99942098	-0.0041787	0.99968493	-5.93E-06	0.99994558	0.04499673	0.68304847	-3.33E-06	0.9999789
ZEB2	0.14887357	0.25651405	2.90E-06	0.99999693	3.66E-06	0.999978	0.02692087	0.13393926	2.47627632	2.84E-04	4.66E-06	0.99987372	0.01279348	0.99206849	1.46374221	0.09906877	0.00663538	0.99968493	1.86E-06	0.99994558	0.16719297	0.33317659	4.34E-06	0.9999789
ZFP57	-0.0050992	NA	-2.32E-07	NA	-6.34E-08	0.999978	-5.00E-04	NA	-7.40E-04	0.99925116	-1.23E-08	0.99987372	-9.98E-04	0.00266257	-1.13E-07	NA	-4.66E-04	NA	9.72E-09	0.99994558	-0.0016117	NA	-9.01E-08	0.9999789
ZNF462	3.65185924	0.00588145	1.90319759	0.17835439	2.37E-06	0.58959497	0.00343684	NA	0.00353397	0.99925116	7.25E-08	0.99987372	NA	NA	-1.36E-07	NA	1.39E-04	NA	NA	NA	-0.0032558	NA	1.57E-07	0.9999789
ZNF608	0.00795419	NA	2.71E-07	NA	1.63E-08	0.999978	NA	NA	NA	NA	4.89E-08	0.99987372	NA	NA	-1.45E-08	NA	1.14E-04	NA	NA	NA	NA	NA	NA	NA
ZNF827	0.9656924	0.07394822	3.58E-06	0.99999693	5.05E-06	0.999978	0.01258234	0.99962555	0.01717233	0.99925116	2.22446049	0.00189944	0.02596608	0.99206849	4.02E-06	0.99942098	0.00603026	0.99968493	2.08E-06	0.99994558	0.04988964	0.78371682	1.78E-06	0.9999789

SYMBOL	Vd1gdT_log2	Vd1gdT_AdjP	Activated_log	Activated_Ad	NK_log2FC	NK_AdjP	BNv_log2FC	BNv_AdjP	BMm_log2FC	BMm_AdjP	Plasma_log2	Plasma_AdjP	ClasMono_lo	ClasMono_A	NClasMono_	NClasMono_	cDC1_log2FC	cDC1_AdjP	cDC2_log2FC	cDC2_AdjP	pDC_log2FC	pDC_AdjP
ANLN	2.01E-08	0.99973907	-1.85E-06	0.99997036	-4.22E-07	0.99991875	8.89E-08	0.99992631	1.10E-07	NA	-2.44E-07	0.99999354	-1.21E-06	0.99977794	-1.67E-07	1.06E-07	NA	NA	-4.21E-07	0.99979039	-1.91E-08	0.9999314
ARID5B	4.41E-06	0.99973907	7.11E-06	0.99997036	2.63E-06	0.99991875	3.32E-06	0.99992631	3.44E-07	0.99968108	1.80E-06	0.99999354	1.66E-06	0.99977794	6.89E-07	0.99998233	-2.23E-07	0.99993076	1.65E-06	0.99979039	1.67E-06	0.9999314
ARRDC3	7.80E-07	0.99973907	-4.20E-07	0.99997036	2.13E-06	0.99991875	1.83E-06	0.99992631	2.22E-06	0.99968108	2.89E-07	0.99999354	9.54E-07	0.99977794	1.80E-06	0.99998233	-4.08E-07	0.99993076	1.06E-07	0.99979039	1.88E-06	0.9999314
ATP6V1A	6.26E-07	0.99973907	-3.81E-07	0.99997036	7.46E-06	0.99991875	8.25E-06	0.99992631	2.73E-06	0.99968108	8.96E-07	0.99999354	-3.02E-07	0.99977794	2.63E-06	0.99998233	2.55E-07	0.99993076	-5.69E-07	0.99979039	1.13E-06	0.9999314
ATXN1	8.72E-07	0.99973907	-4.27E-07	0.99997036	2.23E-06	0.99991875	-2.30E-06	0.99992631	1.14E-09	0.99968108	2.12E-06	0.99999354	1.83E-06	0.99977794	2.98E-06	0.99998233	9.49E-08	0.99993076	1.31E-06	0.99979039	6.02E-07	0.9999314
AVPI1	-1.54E-06	0.99973907	-2.14E-07	0.99997036	-6.68E-06	0.97753683	-3.69E-06	0.99992631	-2.70E-06	0.95589787	-1.69E-07	0.99999354	-1.66E-06	0.99977794	-9.33E-07	0.99998233	-1.14E-07	0.99993076	-3.17E-06	0.99979039	-3.81E-07	0.9999314
B4GALNT3	7.93E-08	0.99973907	7.99E-08	0.99997036	6.66E-07	0.99991875	-7.26E-08	0.99992631	-2.76E-08	NA	2.83E-08	0.99999354	-1.12E-06	0.99977794	4.28E-08	0.99998233	NA	NA	NA	NA	NA	NA
BCL7A	7.98E-07	0.99973907	4.12E-07	0.99997036	1.92E-06	0.99991875	9.61E-07	0.99992631	6.00E-06	0.99968108	-3.75E-07	0.99999354	5.38E-06	0.9966932	-2.51E-07	0.99998233	2.56E-07	0.99993076	-5.22E-06	0.99979039	-1.85E-06	0.9999314
BEND4	-2.10E-09	0.99973907	-5.90E-08	0.99997036	-6.76E-07	0.99991875	-6.11E-07	0.99992631	-1.48E-07	0.99968108	1.61E-07	0.99999354	7.19E-07	0.99977794	2.17E-08	0.99998233	NA	NA	1.04E-08	0.99979039	-2.54E-07	0.9999314
BHLHE40	-6.78E-06	0.05116543	-3.39E-07	0.99997036	-7.31E-07	0.99991875	1.12E-06	0.99992631	1.98E-06	0.99968108	3.41E-07	0.99999354	2.30E-06	0.99977794	3.13E-06	0.99998233	1.02E-06	0.99993076	3.07E-06	0.99979039	1.50E-06	0.9999314
CCDC110	NA	NA	NA	NA	8.22E-08	0.99991875	-4.50E-07	0.99992631	-6.32E-08	0.99968108	1.31E-07	0.99999354	-2.95E-07	NA	NA	NA	NA	NA	NA	NA	NA	NA
CCR6	1.25E-08	0.99973907	-5.75E-07	0.99997036	-6.62E-07	0.99991875	3.12E-06	0.99992631	1.40E-06	0.99968108	-2.91E-08	0.99999354	1.80E-06	0.99977794	4.41E-08	0.99998233	2.86E-07	0.99993076	-6.09E-07	0.99979039	-1.33E-07	0.9999314
CCR7	1.58E-06	0.99973907	9.00E-07	0.99997036	-8.50E-07	0.99991875	-2.49E-07	0.99992631	-8.56E-07	0.99968108	1.24E-08	0.99999354	4.64E-07	0.99977794	-4.70E-07	0.99998233	2.10E-07	0.99993076	1.15E-06	0.99979039	-2.00E-07	0.9999314
CCSER1	2.00E-08	0.99973907	2.84E-08	0.99997036	-1.59E-06	0.99991875	-3.38E-06	0.99992631	-2.4093434	3.47E-04	7.01E-08	0.99999354	1.54E-08	0.99977794	7.55E-07	0.99998233	-2.08E-07	0.99993076	1.19E-07	0.99979039	-4.41E-07	0.9999314
CD8A	-3.61E-07	0.99973907	-1.99E-06	0.99997036	-3.03E-06	0.99991875	-2.1682806	0.12197843	-1.74E-06	0.99968108	-4.53E-08	0.99999354	-2.0351485	0.030925	-4.59E-06	0.99998233	NA	NA	-5.43E-07	0.99979039	-2.25E-07	0.9999314
CD8B	-3.01E-07	0.99973907	-5.67E-06	0.99997036	-4.90E-06	0.99991875	-3.5556392	1.38E-04	-3.10E-06	0.63456812	-2.25E-08	0.99999354	-1.27E-05	1.10E-05	-2.76E-06	0.99998233	NA	NA	-1.70E-06	0.99979039	-1.35E-07	0.9999314
CDC14A	2.62E-06	0.99973907	5.59E-06	0.99997036	7.41E-06	0.99991875	6.66E-06	0.99992631	4.26E-06	0.99968108	-9.20E-07	0.99999354	4.78E-06	0.99977794	1.61E-06	0.99998233	-1.54E-07	0.99993076	3.76E-06	0.99979039	3.52E-06	0.9999314
CHN1	1.02E-07	0.99973907	-1.65E-07	0.99997036	-7.00E-07	0.99991875	-1.19E-07	0.99992631	-8.92E-07	0.99968108	-3.43E-08	0.99999354	-8.00E-07	0.99977794	-6.72E-08	0.99998233	NA	NA	6.83E-08	0.99979039	-5.55E-08	0.9999314
CHST2	1.26E-06	0.99973907	9.41E-07	0.99997036	1.68E-06	0.99991875	9.39E-07	0.99992631	4.75E-07	0.99968108	-8.01E-07	0.99999354	3.68E-07	0.99977794	-5.48E-06	0.99998233	1.10E-08	0.99993076	3.44E-07	0.99979039	-4.03E-07	0.9999314
CYP2C8	NA	NA	NA	NA	3.89E-08	0.99991875	1.03E-07	0.99992631	3.69E-10	NA	NA	NA	4.05E-08	NA	NA	NA	NA	NA	NA	NA	NA	NA
CYP4F12	NA	NA	NA	NA	-2.26E-08	0.99991875	1.80E-07	0.99992631	NA	NA	NA	NA	NA	NA	NA	NA	NA	NA	NA	NA	NA	NA
CYP4V2	-1.21E-07	0.99973907	-1.44E-06	0.99997036	-2.53E-06	0.99991875	-5.32E-06	0.99992631	-5.15E-06	0.95589787	1.04E-08	0.99999354	-3.28E-06	0.99977794	-1.76E-06	0.99998233	-3.34E-07	0.99993076	-1.29E-06	0.99979039	-1.93E-06	0.9999314
DLGAP2	NA	NA	4.54E-08	0.99997036	-9.82E-10	0.99991875	9.45E-10	0.99992631	-1.23E-08	NA	-6.64E-08	0.99999354	-5.7169701	3.20E-06	2.76E-07	0.99998233	2.89E-07	0.99993076	-4.48E-08	0.99979039	NA	NA
DUSP1	-4.45E-06	0.99973907	-5.05E-06	0.99997036	-0.6928204	0.54243917	-5.54E-06	0.99992631	-0.5684452	0.38559221	-3.67E-06	0.99999354	-2.22E-07	0.99977794	-1.81E-06	0.99998233	-6.86E-08	0.99993076	-3.15E-06	0.99979039	-4.23E-06	0.9999314
EIF3E	1.75E-06	0.99973907	6.89E-07	0.99997036	-6.19E-06	0.99991875	-6.25E-06	0.99992631	-7.47E-06	0.99968108	-3.39E-07	0.99999354	-2.44E-06	0.99977794	-4.95E-06	0.99998233	-3.14E-06	0.99993076	-3.14E-06	0.99979039	-3.66E-06	0.9999314
EPHA4	1.54E-07	0.99973907	4.00E-07	0.99997036	1.15E-06	0.99991875	6.81E-07	0.99992631	2.98E-06	0.99968108	-1.33E-08	0.99999354	7.23E-07	0.99977794	4.52E-07	0.99998233	5.40E-08	0.99993076	2.38E-07	0.99979039	9.10E-07	0.9999314
FBXL13	3.60E-08	0.99973907	2.76E-07	0.99997036	-5.03E-07	0.99991875	-1.54E-07	0.99992631	-1.69E-06	0.99968108	-4.85E-08	0.99999354	1.99E-07	0.99977794	-3.73E-07	0.99998233	1.45E-07	0.99993076	-9.20E-08	0.99979039	-4.79E-07	0.9999314
FLNB	8.84E-07	0.99973907	3.57E-07	0.99997036	6.33E-07	0.99991875	-1.92E-06	0.99992631	1.99E-06	0.99968108	-8.96E-08	0.99999354	2.31E-06	0.99977794	4.63E-06	0.99998233	-1.41E-07	0.99993076	1.20E-06	0.99979039	-1.37E-06	0.9999314
GAB2	-1.05E-07	0.99973907	6.43E-07	0.99997036	3.16E-06	0.99991875	2.23E-06	0.99992631	1.01E-06	0.99968108	1.60E-06	0.99999354	1.77E-06	0.99977794	4.53E-06	0.99998233	9.39E-07	0.99993076	3.06E-06	0.99979039	1.95E-06	0.9999314
GADD45A	-2.36E-06	0.99973907	-1.05E-06	0.99997036	-6.13E-06	0.99991875	-3.01E-06	0.99992631	-1.10E-06	0.99968108	3.59E-07	0.99999354	1.17E-06	0.99977794	3.57E-07	0.99998233	5.66E-07	0.99993076	1.13E-06	0.99979039	1.65E-07	0.9999314
GATA3	-3.94E-07	0.99973907	7.81E-07	0.99997036	4.41E-06	0.99991875	-2.01E-06	0.99992631	2.00E-06	0.99968108	1.56E-07	0.99999354	3.18E-07	0.99977794	1.29E-06	0.99998233	4.59E-09	0.99993076	-3.05E-08	0.99979039	1.16E-07	0.9999314
GLIS3	NA	NA	NA	NA	-2.14E-07	0.99991875	-1.70E-06	0.99992631	-7.95E-08	NA	NA	NA	1.52E-07	0.99977794	5.71E-07	0.99998233	NA	NA	-1.16E-07	0.99979039	NA	NA
GPR135	1.68E-08	0.99973907	1.95E-07	0.99997036	-1.26E-06	0.99991875	-1.20E-06	0.99992631	-1.77E-06	0.99968108	1.27E-07	0.99999354	-7.51E-07	0.99977794	6.50E-07	0.99998233	-1.78E-07	0.0027472	6.66E-08	0.99979039	-6.19E-09	0.9999314
GPRIN3	1.94E-06	0.99973907	2.40E-06	0.99997036	6.59E-06	0.99991875	2.10E-06	0.99992631	6.27E-07	0.99968108	2.27E-06	0.99999354	1.81E-06	0.99977794	1.43E-06	0.99998233	-4.20E-07	0.99993076	-6.92E-07	0.99979039	5.43E-08	0.9999314
HDGFL3	1.55E-06	0.99973907	-1.43E-06	0.99997036	9.63E-07	0.99991875	6.20E-07	0.99992631	9.60E-07	0.99968108	9.27E-08	0.99999354	2.00E-06	0.99977794	8.44E-07	0.99998233	-4.02E-07	0.99993076	1.19E-06	0.99979039	-4.00E-07	0.9999314
HERC1	-1.24E-06	0.99973907	9.49E-06	0.99997036	8.94E-06	0.99991875	8.11E-06	0.99992631	8.31E-06	0.99968108	3.80E-06	0.99999354	4.68E-06	0.99977794	4.05E-06	0.99998233	1.32E-06	0.99993076	2.86E-06	0.99979039	7.10E-07	0.9999314
HOXB4	2.23E-07	0.99973907	7.20E-07	0.99997036	5.21E-06	0.99991875	1.76E-06	0.99992631	2.41E-06	0.99968108	1.09E-07	0.99999354	2.10746196	0.15975322	2.07E-06	0.99998233	7.20E-08	0.99993076	4.12E-07	0.99979039	3.54E-07	0.9999314
HSBP1L1	1.35E-07	0.99973907	3.98E-07	0.99997036	-3.64E-06	0.99991875	-6.30E-06	0.8430791	-2.93E-06	0.99968108	5.44E-07	0.99999354	-4.23E-06	0.99977794	-1.29E-07	0.99998233	-5.57E-09	0.99993076	-1.15E-06	0.99979039	-3.15E-07	0.9999314
ID2	-1.30E-06	0.99973907	-6.74E-07	0.99997036	-2.28E-06	0.99991875	-1.73E-06	0.99992631	3.40E-06	0.99968108	1.79E-08	0.99999354	-8.66E-06	0.99977794	9.74E-07	0.99998233	4.62E-08	0.99993076	-5.41E-06	0.99979039	1.21E-06	0.9999314
IGF2BP2	2.79E-07	0.99973907	-5.82E-07	0.99997036	-8.98E-07	0.99991875	-1.91E-07	0.99992631	-2.84E-07	NA	5.76E-08	0.99999354	3.19E-06	0.99977794	4.22E-07	0.99998233	-5.66E-07	0.99993076	1.01E-06	0.99979039	-1.82E-06	0.9999314
IGF2BP3	1.93E-07	0.99973907	1.35E-07	0.99997036	1.42E-06	0.99991875	2.85944433	0.20440375	2.91207444	0.00158458	2.52E-08	0.99999354	2.81E-06	0.99977794	6.11E-07	0.99998233	1.05E-07	0.99993076	2.56E-07	0.99979039	1.90E-07	0.9999314
IGFBP3	NA	NA	NA	NA	-1.26E-06	0.99991875	-2.41E-07	0.99992631	5.26E-08	NA	NA	NA	-7.53E-07	0.99977794	NA	NA	NA	NA	-1.20E-08	0.99979039	-3.68E-07	0.9999314
IKZF2	4.18E-06	0.99973907	1.91E-06	0.99997036	1.76E-06	0.99991875	6.88E-06	0.99992631	1.31E-06	0.99968108	1.02E-07	0.99999354	1.70E-06	0.99977794	8.10E-07	0.99998233	9.50E-08	0.99993076	2.14E-07	0.99979039	1.16E-06	0.9999314
IL3	NA	NA	NA	NA	-2.02E-07	0.99991875	3.01E-08	0.99992631	NA	NA	NA	NA	-8.93E-08	0.00588247	NA	NA	NA	NA	NA	NA	NA	NA
JAKMIP2	5.93E-07	0.99973907	1.28E-06	0.99997036	6.95E-07	0.99991875	2.14E-06	0.99992631	4.76E-07	0.99968108	-3.21E-08	0.99999354	1.36E-06	0.99977794	1.82E-06	0.99998233	-3.17E-08	0.99993076	-4.43E-07	0.99979039	4.78E-07	0.9999314
KIR3DL2	-4.13E-07	0.99973907	1.78E-06	0.99997036	-2.61E-06	0.99991875	-4.91E-07	0.99992631	1.02E-07	NA	2.35E-07	0.99999354	-1.73E-06	0.99977794	2.25E-07	0.99998233	NA	NA	1.38E-07	0.99979039	NA	NA
KLF6	2.94E-06	0.99973907	2.24E-06	0.99997036	1.72E-06	0.99991875	3.68E-06	0.99992631	3.52E-06	0.99968108	-1.97E-06	0.99999354	5.37E-07	0.99977794	1.39E-06	0.99998233	1.25E-06	0.99993076	-3.18E-07	0.99979039	2.88E-06	0.9999314
KLRF1	-1.88E-06	0.99973907	-3.72E-06	0.99997036	-3.20E-06	0.99991875	-2.27E-06	0.99992631	-1.07E-06	0.99968108	9.43E-08	0.99999354	-4.94E-06	0.99977794	-5.54E-07	0.99998233	1.47E-06	0.99993076	-6.71E-07	0.99979039	1.96E-07	0.9999314
KRT18	NA	NA	3.25E-08	0.99997036	-5.56E-07	0.99991875	-3.35E-06	0.99992631	-9.74E-08	0.99968108	7.09E-08	0.99999354	-5.50E-07	0.99977794	-9.36E-08	0.99998233	-2.38E-08	0.99993076	-7.52E-07	0.99979039	9.28E-08	0.9999314
KRT72	2.27E-07	0.99973907	1.83E-08	0.99997036	-2.84E-06	0.99991875	-6.57E-07	0.99992631	-1.44E-07	NA	NA	NA	-2.28E-07	0.99977794	-8.51E-08	0.99998233	NA	NA	NA	NA	NA	NA
LGALS1	-1.14E-06	0.99973907	-3.59E-07	0.99997036	1.43E-06	0.99991875	-7.18E-07	0.99992631	-4.62E-06	0.99968108	-1.71E-06	0.99999354	3.83E-06	0.99977794	3.04E-06	0.99998233	7.14E-07	0.99993076	2.42E-06	0.99979039	2.14E-06	0.9999314
LGALS3	8.84E-08	0.99973907	4.32E-07	0.99997036	-3.82E-06	0.99991875	7.45E-07	0.99992631	1.29E-06	0.99968108	-4.31E-06	0.99999354	2.67E-06	0.99977794	7.66E-08	0.99998233	2.26E-07	0.99993076	2.73E-06	0.99979039	-7.94E-07	0.9999314
LIN7A	NA	NA	-1.35E-07	0.02408674	-9.15E-07	0.99991875	-6.39E-08	0.99992631	8.07E-08	NA	NA	NA	-1.5924099	0.00136391	-2.50E-06	0.99998233	NA	NA	NA	NA	NA	NA
LYAR	-1.84E-06	0.99973907	-9.34E-07	0.99997036	-7.95E-07	0.99991875	-5.19E-07	0.99992631	2.49E-06	0.99968108	2.34E-07	0.99999354	-2.32E-06	0.99977794	-1.80E-06	0.99998233	1.49E-07	0.99993076	-1.03E-06	0.99979039	5.07E-08	0.9999314
LYZ	1.05E-06	0.99973907	-2.78E-06	0.99997036	-1.0733405	0.25082422	-0.9789759	0.75715857	-7.88E-06	0.51608858	-3.28E-07	0.99999354	-1.41E-05	0.6810435	-5.46E-06	0.99998233	4.43E-08	0.99993076	-1.55E-05	0.99979039	-9.81E-07	0.9999314
MGAT4A	-1.98E-06	0.99973907	-1.89E-07	0.99997036	-5.44E-06	0.99991875	-5.44E-06	0.99992631	2.24E-06	0.99968108	9.36E-07	0.99999354	-1.40E-07	0.99977794	4.41E-07	0.99998233	-5.51E-07	0.99993076	-3.24E-06	0.99979039	-1.54E-06	0.9999314
MME	4.86E-08	0.99973907	1.59E-07	0.99997036	9.77E-07	0.99991875	6.14E-07	0.99992631	-3.26E-10	0.99968108	6.04E-08	0.99999354	1.22E-06	0.99977794	4.00E-08	0.99998233	NA	NA	8.67E-08	0.99979039	-8.03E-07	0.9999314
MMP28	NA	NA	NA	NA	-6.01E-07	0.99991875	-2.03E-06	0.99992631	-2.60E-07	0.99968108	NA	NA	-9.14E-07	0.99977794	-1.58E-07	0.99998233	NA	NA	-1.85E-07	0.99979039	-1.36E-07	0.9999314
MOB1B	1.13E-06	0.99973907	6.92E-07	0.99997036	6.10E-06	0.99991875	3.74E-06	0.99992631	1.2642101	0.27235636	1.71E-08	0.99999354	6.44E-06	0.99977794	8.36E-07	0.99998233	9.20E-07	0.99993076	2.17E-06	0.99979039	2.88E-06	0.9999314
MPDZ	NA	NA	NA	NA	-1.45E-07	0.99991875	NA	NA	3.80E-08	NA	NA	NA	1.53E-07	0.99977794	NA	NA	-1.81E-07	0.00274618	NA	NA	5.21E-08	0.9999314
MYC	9.02E-07	0.99973907	1.45E-06	0.99997036	1.38E-06	0.99991875	3.69E-07	0.99992631	4.21E-06	0.99968108	1.93E-07	0.99999354	2.27E-06	0.99977794	1.99E-06	0.99998233	-3.19E-08	0.99993076	1.80E-06	0.99979039	2.69E-07	0.9999314
MZB1	3.20E-07	0.99973907	4.72E-07	0.99997036	1.59E-06	0.99991875	-2.07E-07	0.99992631	1.68E-06	0.99968108	1.86E-07	0.99999354	2.47E-06	0.99977794	8.81E-07	0.99998233	-3.93E-08	0.99993076	1.50E-07	0.99979039	-9.65E-07	0.9999314
NCL	-4.00E-06	0.99973907	-4.99E-06	0.99997036	-6.81E-06	0.99991875	-2.32E-06	0.99992631	-2.64E-06	0.99968108	-4.56E-06	0.99999354	7.03E-07	0.99977794	-7.93E-06	0.99998233	-1.23E-06	0.99993076	-2.51E-06	0.99979039	-7.88E-06	0.9999314
NDFIP1	1.09E-06	0.99973907	-3.38E-06	0.99997036	-1.15E-06	0.99991875	-6.32E-06	0.99992631	-5.14E-06	0.99968108	1.74E-06	0.99999354	-5.80E-06	0.99977794	-1.99E-06	0.99998233	-1.79E-06	0.99993076	-2.92E-06	0.99979039	-7.55E-07	0.9999314
NEFL	1.37E-08	0.99973907	NA	NA	5.55E-09	0.99991875	-8.79E-08	0.99992631	NA	NA	NA	NA	-1.02E-07	0.99977794	NA	NA	NA	NA	NA	NA	NA	NA
NEGR1	-2.17E-07	0.99973907	1.25E-07	0.99997036	-6.85E-07	0.99991875	-9.32E-08	0.99992631	1.30E-07	NA	NA	NA	-2.97E-07	0.99977794	-2.01E-07	0.99998233	-2.21E-07	0.99993076	-5.08E-07	0.99979039	-1.77E-06	0.9999314
NPDC1	-7.71E-07	0.99973907	2.18E-07	0.99997036	9.48E-07	0.99991875	-3.95E-07	0.99992631	1.25E-07	0.99968108	1.02E-07	0.99999354	2.79E-06	0.99977794	5.58E-07	0.99998233	-6.02E-08	0.99993076	-1.43E-07	0.99979039	-1.36E-07	0.9999314
NR3C2	4.13E-07	0.99973907	-4.31E-07	0.99997036	-4.94E-06	0.97753683	-2.45E-06	0.99992631	-4.36E-06	0.56245273	6.27E-08	0.99999354	-2.29E-06	0.99977794	-9.73E-07	0.99998233	-2.46E-08	0.99993076	-6.56E-07	0.99979039	7.79E-09	0.9999314
NRP2	3.57E-08	0.99973907	-4.34E-08	0.99997036	1.63E-07	0.99991875	6.47E-07	0.99992631	1.13E-06	0.99968108	NA	NA	1.28E-06	0.99977794	1.51E-06	0.99998233	4.70E-07	0.99993076	1.46E-06	0.99979039	NA	NA
NTRK2	NA	NA	NA	NA	-1.59E-08	0.99991875	3.79E-08	0.99992631	6.58E-08	NA	NA	NA	-9.34E-08	NA	NA	NA	NA	NA	3.92E-08	0.99979039	NA	NA
PARD3	3.05E-07	0.99973907	1.03E-07	0.99997036	3.51E-07	0.99991875	6.96E-07	0.99992631	3.55E-07	NA	NA	NA	1.41E-07	0.99977794	-6.39E-08	0.99998233	NA	NA	7.96E-08	0.99979039	-2.80E-08	0.9999314
PCBP3	9.59E-08	0.99973907	-9.88E-08	0.99997036	8.62E-07	0.99991875	-2.23E-07	0.99992631	-2.0983567	0.00158458	5.35E-08	0.99999354	1.98E-06	0.99977794	2.14E-06	0.99998233	1.33E-09	0.99993076	8.88E-07	0.99979039	6.82E-08	0.9999314
PDLIM5	6.44E-07	0.99973907	-8.61E-08	0.99997036	2.75E-06	0.99991875	4.92E-07	0.99992631	4.54E-07	0.99968108	3.62E-07	0.99999354	6.79E-06	0.98433151	4.67E-06	0.99998233	5.62E-07	0.99993076	7.17E-06	0.99979039	-1.25E-07	0.9999314
PHLDA1	1.56E-07	0.99973907	1.72E-07	0.99997036	-1.66E-07	0.99991875	1.04E-06	0.99992631	1.95E-06	0.99968108	1.24E-07	0.99999354	9.53E-07	0.99977794	2.09E-06	0.99998233	1.92E-07	0.99993076	1.06E-06	0.99979039	1.31E-07	0.9999314
PHLDB2	4.39E-07	0.99973907	-1.33E-06	0.99997036	1.58E-06	0.99991875	1.24E-06	0.99992631	-4.40E-07	0.99968108	1.40E-07	0.99999354	1.20E-06	0.99977794	8.45E-07	0.99998233	-1.65E-07	0.99993076	7.18E-07	0.99979039	2.42E-07	0.9999314
PI16	-4.02E-08	0.99973907	-7.38E-07	0.99997036	6.97E-07	0.99991875	-5.09E-08	0.99992631	2.89E-08	NA	NA	NA	-4.46E-07	0.99977794	-2.54E-08	0.99998233	4.67E-08	0.99993076	3.29E-07	0.99979039	NA	NA
PIK3C2B	6.26E-07	0.99973907	1.21E-06	0.99997036	4.33E-06	0.99991875	1.45E-06	0.99992631	4.88E-06	0.99968108	3.44E-07	0.99999354	3.78E-06	0.99977794	4.12E-07	0.99998233	NA	NA	-2.87E-07	0.99979039	-7.82E-08	0.9999314
PLAAT3	-5.63E-07	0.99973907	3.32E-06	0.99997036	3.18E-06	0.99991875	3.98E-08	0.99992631	2.29E-06	0.99968108	2.15E-06	0.99999354	5.63E-06	0.99977794	4.54E-06	0.99998233	-1.88E-08	0.99993076	-6.65E-07	0.99979039	-1.58E-06	0.9999314
PLCL1	7.14E-07	0.99973907	-5.53E-07	0.99997036	-4.53E-06	0.13285952	-5.05E-06	0.99992631	-1.09E-06	0.99968108	5.12E-08	0.99999354	-4.95E-06	0.97781721	-1.82E-06	0.99998233	3.37E-07	0.99993076	-6.16E-07	0.99979039	-1.22E-07	0.9999314
PLD6	2.59E-08	0.99973907	-3.42E-07	0.99997036	-2.25E-06	0.68693727	-2.86E-06	0.99992631	-3.26E-06	0.99968108	5.95E-08	0.99999354	-8.92E-07	0.99977794	-6.01E-07	0.99998233	NA	NA	-6.71E-09	0.99979039	-9.13E-08	0.9999314
PRICKLE2	NA	NA	NA	NA	3.84E-07	0.99991875	3.98E-08	0.99992631	-1.93E-07	0.99968108	9.51E-08	0.99999354	-2.57E-07	0.99977794	NA	NA	NA	NA	NA	NA	NA	NA
PTCH2	-3.46E-07	0.99973907	-9.25E-08	0.99997036	-1.29E-06	0.99991875	-1.14E-06	0.99992631	-1.63E-06	0.99968108	-1.91E-07	0.99999354	-1.24E-06	0.99977794	-1.71E-06	0.99998233	-5.32E-08	0.99993076	-7.56E-08	0.99979039	-8.30E-07	0.9999314
PTPN13	NA	NA	3.44E-07	0.99997036	-1.06E-07	0.99991875	-5.83E-07	0.99992631	1.25E-07	NA	NA	NA	6.27E-08	0.99977794	-1.25E-06	0.99998233	NA	NA	1.18E-07	0.99979039	NA	NA
RADX	-9.30E-07	0.99973907	-1.12E-06	0.99997036	-3.15E-06	0.99991875	-2.51E-06	0.99992631	-2.62E-06	0.99968108	5.25E-08	0.99999354	-2.67E-06	0.99977794	-3.48E-07	0.99998233	-1.04E-07	0.99993076	-4.04E-07	0.99979039	3.91E-07	0.9999314
RAPGEF1	-6.48E-07	0.99973907	3.18E-06	0.99997036	3.60E-06	0.99991875	4.45E-06	0.99992631	6.09E-06	0.99968108	-2.04E-07	0.99999354	2.33E-06	0.99977794	5.66E-06	0.99998233	1.28E-06	0.99993076	3.04E-06	0.99979039	8.44E-07	0.9999314
RBBP8	5.38E-07	0.99973907	-6.24E-07	0.99997036	2.17E-06	0.99991875	3.32E-06	0.99992631	2.86E-06	0.99968108	-6.42E-07	0.99999354	-8.87E-07	0.99977794	-1.42E-06	0.99998233	-1.16E-07	0.99993076	1.89E-06	0.99979039	-4.97E-07	0.9999314
RFX2	2.81E-07	0.99973907	6.80E-07	0.99997036	2.52E-06	0.99991875	3.73E-06	0.99992631	3.60E-06	0.99968108	1.39E-07	0.99999354	3.17E-06	0.94349148	2.20E-06	0.99998233	3.93E-07	0.99993076	2.79E-06	0.99979039	-7.92E-08	0.9999314
RIC3	5.55E-07	0.99973907	-7.00E-07	0.99997036	-4.51E-06	0.99991875	-6.58E-06	0.99992631	-6.52E-06	0.99968108	-1.07E-06	0.99999354	-2.34E-06	0.99977794	-2.05E-06	0.99998233	-2.82E-07	0.99993076	-7.02E-07	0.99979039	1.63E-07	0.9999314
RNF175	NA	NA	NA	NA	-1.29E-07	0.99991875	-7.14E-07	0.99992631	1.30E-08	NA	NA	NA	-2.81E-06	0.99977794	-9.42E-07	0.99998233	NA	NA	-5.75E-08	0.99979039	NA	NA
RORB	3.44E-07	0.99973907	1.13E-07	0.99997036	3.69E-06	0.12118599	8.96E-07	0.99992631	3.15E-07	NA	NA	NA	5.72E-07	0.99977794	2.36E-09	0.99998233	NA	NA	NA	NA	-1.23E-07	0.9999314
RTKN2	2.01E-06	0.99973907	3.30484172	1.22E-08	2.83718889	0.13285952	1.24153093	0.84644274	2.57963494	0.63316758	6.81E-08	0.99999354	1.858729	0.3191258	1.40E-06	0.99998233	1.17E-07	0.99993076	5.74E-07	0.99979039	9.76E-08	0.9999314
S100B	5.95E-08	0.99973907	7.16E-07	0.99997036	-6.28E-08	0.99991875	-1.66E-06	0.99992631	-4.93E-07	0.99968108	-4.15E-08	0.99999354	3.18E-08	0.99977794	9.91E-08	0.99998233	-1.41E-07	0.99993076	-7.20E-07	0.99979039	-7.41E-08	0.9999314
SAMD12	3.72E-07	0.99973907	-4.65E-07	0.99997036	-2.18E-06	0.90548278	-3.74E-06	0.00569603	-2.85E-06	0.53127752	-7.81E-08	0.99999354	-2.67E-06	0.9966932	-9.92E-07	0.99998233	1.97E-08	0.99993076	-4.52E-07	0.99979039	-3.54E-07	0.9999314
SESTD1	5.26E-07	0.99973907	-6.61E-07	0.99997036	2.75E-06	0.99991875	1.95E-06	0.99992631	3.82E-06	0.99968108	2.42E-07	0.99999354	-1.31E-06	0.99977794	3.11E-06	0.99998233	-4.10E-07	0.99993076	9.32E-09	0.99979039	6.07E-07	0.9999314
SIPA1L2	5.13E-07	0.99973907	1.60E-07	0.99997036	-1.21E-06	0.99991875	8.32E-07	0.99992631	-7.31E-07	NA	3.36E-08	0.99999354	1.34E-06	0.99977794	-1.24E-06	0.99998233	NA	NA	7.03E-07	0.99979039	1.35E-07	0.9999314
SLC22A17	7.54E-08	0.99973907	3.62E-08	0.99997036	-1.18E-06	0.99991875	-6.14E-07	0.99992631	-5.08E-07	NA	NA	NA	-1.40E-06	0.99977794	-7.72E-09	0.99998233	NA	NA	NA	NA	-9.72E-08	0.9999314
SLC37A3	8.60E-07	0.99973907	2.04E-06	0.99997036	2.71E-06	0.99991875	3.43E-06	0.99992631	3.78E-06	0.80426533	1.48E-07	0.99999354	3.70E-06	0.99977794	2.82E-06	0.99998233	1.04E-07	0.99993076	1.27E-06	0.99979039	6.67E-07	0.9999314
SLC7A4	NA	NA	NA	NA	NA	NA	NA	NA	NA	NA	NA	NA	NA	NA	NA	NA	NA	NA	NA	NA	NA	NA
SMC4	2.86578072	0.99973907	-1.76E-06	0.99997036	7.42E-06	0.99991875	7.60E-06	0.99992631	5.97E-06	0.99968108	-3.37E-06	0.99999354	4.74E-07	0.99977794	-1.16E-06	0.99998233	1.68E-07	0.99993076	1.06E-06	0.99979039	7.40E-07	0.9999314
SNED1	3.44E-07	0.99973907	-4.28E-07	0.99997036	5.96E-08	0.99991875	-3.71E-07	0.99992631	4.16E-08	0.99968108	5.50E-08	0.99999354	-3.13E-07	0.99977794	-5.48E-06	0.99998233	NA	NA	5.28E-07	0.99979039	-8.05E-09	0.9999314
SNRK	-2.13E-06	0.99973907	-6.50E-07	0.99997036	-4.43E-07	0.99991875	-6.26E-06	0.99992631	-2.00E-06	0.99968108	1.03E-06	0.99999354	-2.69E-06	0.99977794	-1.46E-06	0.99998233	2.12E-07	0.99993076	-5.92E-07	0.99979039	-9.62E-07	0.9999314
SOX4	7.47E-08	0.99973907	1.61E-06	0.99997036	3.22E-06	0.99991875	2.54E-06	0.99992631	1.67089936	0.04077451	1.63E-07	0.99999354	2.62E-06	0.99977794	-1.11E-06	0.99998233	1.77E-06	0.99993076	2.65E-06	0.99979039	2.18E-06	0.9999314
SUMO4	2.03E-07	0.99973907	1.09E-06	0.99997036	4.93E-07	0.99991875	3.27E-06	0.99992631	2.16E-06	0.99968108	1.95E-07	0.99999354	1.80E-06	0.99977794	9.33E-07	0.99998233	2.12E-07	0.99993076	1.47E-06	0.99979039	1.93E-06	0.9999314
TAB2	-4.08E-08	0.99973907	2.87E-06	0.99997036	4.46E-06	0.99991875	5.93E-06	0.99992631	5.49E-06	0.99968108	2.73E-06	0.99999354	2.26E-06	0.99977794	3.35E-06	0.99998233	1.76E-07	0.99993076	4.35E-06	0.99979039	6.16E-06	0.9999314
TBX3	1.07E-06	0.99973907	NA	NA	NA	NA	6.97E-08	0.99992631	NA	NA	NA	NA	5.22695643	0.00136391	5.11E-08	0.99998233	NA	NA	1.96E-07	0.99979039	NA	NA
TCEAL2	NA	NA	NA	NA	-1.49E-07	0.99991875	NA	NA	-1.42E-07	NA	NA	NA	-2.59E-07	0.99977794	NA	NA	NA	NA	NA	NA	NA	NA
TMEM220	1.27E-08	0.99973907	5.98E-08	0.99997036	6.55E-07	0.99991875	-8.83E-07	0.99992631	-1.16E-06	0.99968108	-9.84E-09	0.99999354	7.96E-07	0.99977794	3.92E-07	0.99998233	-3.10E-07	0.99993076	-3.32E-07	0.99979039	3.02E-08	0.9999314
TNFSF10	1.42E-06	0.99973907	-2.15E-08	0.99997036	5.74E-06	0.99991875	1.49E-06	0.99992631	2.32E-06	0.99968108	9.22E-07	0.99999354	7.11E-07	0.99977794	2.21E-06	0.99998233	4.33E-07	0.99993076	6.24E-07	0.99979039	-7.03E-07	0.9999314
TOX2	1.68E-06	0.99973907	1.47E-06	0.99997036	1.39E-06	0.99991875	1.42E-06	0.99992631	1.03E-06	0.99968108	4.92E-07	0.99999354	1.56E-06	0.99977794	-1.85E-07	0.99998233	NA	NA	1.64E-07	0.99979039	1.39E-07	0.9999314
TP53INP1	7.95E-07	0.99973907	2.16E-06	0.99997036	6.12E-06	0.99991875	-4.57E-06	0.99992631	-1.62E-06	0.99968108	1.18E-07	0.99999354	6.23E-07	0.99977794	1.15E-06	0.99998233	-9.52E-08	0.99993076	-1.28E-06	0.99979039	3.70E-08	0.9999314
TRIO	1.32E-06	0.99973907	2.78E-07	0.99997036	1.72E-06	0.99991875	-1.98E-07	0.99992631	-1.30E-06	0.99968108	4.65E-07	0.99999354	6.59E-06	0.99977794	1.89E-06	0.99998233	2.84E-07	0.99993076	3.77E-07	0.99979039	1.45E-06	0.9999314
TRMO	-3.29E-07	0.99973907	2.16E-07	0.99997036	-3.25E-06	0.99991875	-1.10E-05	0.63054231	-5.17E-06	0.99968108	9.99E-08	0.99999354	-1.95E-06	0.99977794	-2.23E-06	0.99998233	-1.22E-08	0.99993076	-5.69E-07	0.99979039	-1.47E-06	0.9999314
TSC22D3	-2.63E-06	0.99973907	-1.64E-06	0.99997036	-9.06E-06	0.99991875	-4.79E-06	0.99992631	-1.61E-05	0.51608858	2.01E-06	0.99999354	2.20E-06	0.99977794	7.60E-07	0.99998233	-8.16E-07	0.99993076	1.21E-06	0.99979039	-1.26E-05	0.9999314
TSHZ2	1.89E-07	0.99973907	-4.44E-07	0.99997036	-7.08E-06	0.17115961	-5.65E-06	0.63054231	-2.82E-07	0.99968108	-1.02E-07	0.99999354	-2.66E-06	0.99977794	-1.30E-06	0.99998233	NA	NA	-1.64E-06	0.99979039	1.21E-08	0.9999314
TTC12	-6.70E-08	0.99973907	-3.42E-07	0.99997036	-2.48E-06	0.99991875	-3.23E-06	0.99992631	-2.84E-06	0.99968108	3.81E-08	0.99999354	-1.50E-06	0.99977794	-2.34E-06	0.99998233	4.73E-08	0.99993076	-5.86E-07	0.99979039	-5.19E-07	0.9999314
UTS2	2.01E-07	0.99973907	-4.24E-07	0.99997036	-4.23E-06	0.27637153	-2.19E-07	0.99992631	-2.25E-07	0.99968108	-1.03E-07	0.99999354	-8.40E-07	0.99977794	-3.48E-07	0.99998233	NA	NA	-1.16E-06	0.99979039	-2.95E-07	0.9999314
VANGL1	3.27E-07	0.99973907	-3.87E-07	0.99997036	3.97E-07	0.99991875	7.76E-07	0.99992631	2.63E-07	NA	1.29E-07	0.99999354	1.00E-06	0.99977794	-5.88E-07	0.99998233	NA	NA	-3.99E-07	0.99979039	2.05E-07	0.9999314
VAV3	3.40E-07	0.99973907	1.08E-06	0.99997036	9.85E-06	0.99991875	1.94E-06	0.99992631	3.26E-06	0.99968108	9.14E-08	0.99999354	2.94E-06	0.99977794	3.02E-06	0.99998233	1.91E-07	0.99993076	8.20E-06	0.99979039	-1.03E-06	0.9999314
WNT7A	NA	NA	NA	NA	-3.69E-07	0.99991875	-2.07E-07	0.99992631	-6.30E-08	NA	NA	NA	-1.43E-06	0.99977794	-3.08E-07	0.99998233	NA	NA	-1.65E-08	0.99979039	NA	NA
YBX3	2.15E-06	0.99973907	-5.98E-08	0.99997036	-6.76E-07	0.99991875	-4.08E-06	0.99992631	4.26E-06	0.95589787	-3.49E-06	0.99999354	-9.16E-07	0.99977794	8.22E-07	0.99998233	-3.09E-06	0.99993076	-2.29E-06	0.99979039	-1.81E-06	0.9999314
ZEB2	-1.58E-06	0.99973907	1.91E-06	0.99997036	4.67E-06	0.99991875	8.29E-06	0.99992631	1.11E-05	0.4980572	7.97E-07	0.99999354	2.48E-06	0.99977794	2.51E-06	0.99998233	2.16E-06	0.99993076	6.34E-06	0.99979039	-5.46E-07	0.9999314
ZFP57	NA	NA	-1.59E-07	0.99997036	-2.86E-07	0.99991875	-6.54E-08	0.99992631	-2.14E-08	NA	NA	NA	-1.91E-07	0.99977794	1.28E-08	0.99998233	NA	NA	-9.74E-08	0.99979039	NA	NA
ZNF462	4.92E-08	0.99973907	9.06E-07	0.99997036	1.43E-06	0.93494637	2.46E-07	0.99992631	5.22E-08	NA	NA	NA	1.90E-07	0.99977794	NA	NA	NA	NA	4.77E-08	0.99979039	4.99E-08	0.9999314
ZNF608	NA	NA	2.71E-08	0.99997036	4.21E-07	0.99991875	1.08112745	0.63054231	2.44637909	0.00158458	1.28E-07	0.99999354	1.04E-06	0.99977794	1.57E-07	0.99998233	1.67E-07	0.99993076	2.10E-07	0.99979039	-1.57E-07	0.9999314
ZNF827	5.53E-09	0.99973907	2.05E-06	0.99997036	5.46E-06	0.99991875	2.42E-06	0.99992631	4.60E-06	0.99968108	2.81E-07	0.99999354	3.28E-06	0.99977794	6.07E-07	0.99998233	2.24E-07	0.99993076	5.40E-07	0.99979039	3.64E-07	0.9999314
